# Performance of French medico-administrative databases in epidemiology of infectious diseases: a scoping review

**DOI:** 10.3389/fpubh.2023.1161550

**Published:** 2023-05-12

**Authors:** Marc-Florent Tassi, Nolwenn le Meur, Karl Stéfic, Leslie Grammatico-Guillon

**Affiliations:** ^1^INSERM U1259, Université de Tours, Tours, France; ^2^Univ Rennes, EHESP, CNRS, Inserm, Arènes-UMR 6051, RSMS-U 1309, Rennes, France; ^3^Laboratoire de virologie et CNR VIH-Laboratoire associé, CHRU de Tours, Tours, France; ^4^Service d'Information Médicale d'Epidémiologie et d'Economie de la Santé, CHRU de Tours, Tours, France

**Keywords:** infectious diseases, medico-administrative data, SNDS, validation studies, capture recapture studies, scoping review

## Abstract

The development of medico-administrative databases over the last few decades has led to an evolution and to a significant production of epidemiological studies on infectious diseases based on retrospective medical data and consumption of care. This new form of epidemiological research faces numerous methodological challenges, among which the assessment of the validity of targeting algorithm. We conducted a scoping review of studies that undertook an estimation of the completeness and validity of French medico-administrative databases for infectious disease epidemiological research. Nineteen validation studies and nine capture-recapture studies were identified. These studies covered 20 infectious diseases and were mostly based on the evaluation of hospital claimed data. The evaluation of their methodological qualities highlighted the difficulties associated with these types of research, particularly those linked to the assessment of their underlying hypotheses. We recall several recommendations relating to the problems addressed, which should contribute to the quality of future evaluation studies based on medico-administrative data and consequently to the quality of the epidemiological indicators produced from these information systems.

## 1. Introduction

Major epidemics of the last thirty years, such as HIV, Ebola, or SARS, have brought epidemiology back to its historical link with infectious diseases (1–3). The recent pandemic of COVID-19 has dramatically increased this phenomenon (4–6). Indeed, a simple search on PubMed for “epidemiology” and “infection” returned an annual average of 37,600 references for the years 2015 to 2019 vs. 72,800 for 2020 and 2021.

From a methodological perspective, the last decades have also strongly affected epidemiological research. The rise of Big Data during the information age has notably materialized in the health sector through the progressive implementation of medico-administrative information systems. These data warehouses may contain various medical and demographic information but share the common feature of being fed in a passive, regular and sustainable way for administrative and financial management purposes (7, 8). In France, public health care agencies collect and monitor health care expenses using two principal data warehouses. The nature and scope of the information they contain has already been extensively detailed (9, 10). Briefly, the *Datamart de Consommation Inter Régime* (DCIR), gathers information on primary care expenditures whereas the *Programme de Médicalisation des Systèmes d'Information* (PMSI), relates to hospital care including information extracted from anonymous discharge summaries. The *Système National des Données de Santé* (SNDS) was conceived to host and link these two data warehouses so that they can be used jointly for research purposes. Although these information systems were initially developed for financial management purposes, their content in medical data covering 99% of the French population associated with significant historical depth has made them increasingly valuable as data sources for public health research and in particular epidemiology (11).

The growing success of administrative medical databases for research purposes should not blind researchers to the fact that these sources of information have many limitations that are likely to cause significant bias (7, 12–14). A major concern is the lack of clinical and biological information available which sometimes limits the accuracy and questions the reliability of the information used to identify population of interest. To overcome this limitation, researchers develop algorithms of varying complexity that aim to minimize patient misclassification, whether in terms of inclusion criteria, exposure, comorbidities, or outcome.

In France, users of French medico-administrative databases have formed the REDSIAM network to mutualise expertise concerning the development and evaluation of algorithms for epidemiological purposes (15). In 2017, its “infectious diseases” working group published a narrative review on infections studied through French medico-administrative databases and on the characteristics of the algorithms developed and/or used to identify patients with these infections (16). However, the performances of these algorithms remain a major concern for epidemiological studies based these databases as only few have reported a validation process using a gold standard and their methodological process was never evaluated.

To assess both the validity of French medico-administrative databases for epidemiological purposes in infectious diseases research and the methodological quality of studies that conducted validation of infectious diseases identification algorithms, we undertook a scoping review with the following specific objectives: (1) identify topics where efforts have been made to assess the completeness and validity of these databases; (2) identify and describe the methods and resources used to carry out these validations.

## 2. Methods

We undertook this review based on the PRISMA extension for Scoping Review (PRISMA-ScR) guidelines (17). The protocol of this study was not registered.

### 2.1. Types of study considered in this review

To address the objectives of this review, two methodological frameworks were considered: validation study and capture-recapture study.

Validation studies are commonly used in medical sciences to evaluate the predictive ability of screening and diagnostic procedures by comparing the predictions of these tests to a reference classification. Since diagnostic tests can be conceptually assimilated to classification algorithms, the methodology used to evaluate them can be transposed in a quasi-identical manner to the analysis of the performance of disease targeting algorithms in medical-administrative databases.

The capture-recapture method was originally developed in the field of ecology to estimate the size of animal populations. Adapted to epidemiology, its principle is to cross-reference several databases derived from the same population and containing information on diseased individuals in order to identify common cases. Using the number of cases reported by each source and the number of common cases, it is possible under certain conditions to estimate the total number of affected individuals in the source population and thus the completeness of each database. If one of the databases involved is considered to be exhaustive and the identification of cases within it is based on an algorithmic procedure, then the estimate of its completeness derived from the capture-recapture procedure can be interpreted as the sensitivity of the targeting algorithm.

### 2.2. Search strategy

We searched PubMed, Embase and Web of Science for articles published in English or French up to the end of 2021. To identify relevant studies, our search strategy consisted in associating the concepts “infectious disease” and “French medico-administrative database” using the Boolean operator “AND” (Supplementary 1). Since the terminology used to refer to French medico-administrative databases is not always explicit, we used a broad search lexicon to ensure the identification of studies as complete as possible (18). We also searched for articles in the documentary databases of three French institutions routinely using the SNDS (*Assurance Maladie, Santé publique France, EPI-PHARE*) (19–21).

The concepts of algorithm validation and database completeness assessment were not integrated into the search algorithm to avoid missing studies where these would have consisted in secondary objectives.

### 2.3. Studies selection

To be considered for inclusion in the study, articles had to satisfy four criteria: (1) to follow the “Introduction, Methods, Results, and Discussion” structure, (2) inclusion criteria and/or the main objective of the study directly related to an infectious disease and/or an anti-infective agent, (3) data used had to originate at least partly from a French medico-administrative database, (4) the study had to include at least one evaluative aspect either related to the completeness of the information sources via a capture-recapture method; or related to the performances of the algorithm employed to define the infectious disease and/or the anti-infective drug of interest.

For validation studies, we only considered research comparing medico-administrative data to a reference standard at the individual-patient level. Ecological validations (i.e., comparisons of aggregate statistics across studies) were exclude as they do not allow the calculation of algorithms accuracy indicators and carry too high risk of bias (22).

Using Google Scholar, we reviewed the bibliographies and citations of the articles that satisfied the first three inclusion criteria to find additional research papers of interest.

Abstracts were excluded from analysis.

### 2.4. Data extraction and quality assessment

For studies meeting at least the first three inclusion criteria, we used a standardized abstraction form to describe the research scope and methods: (1) condition of interest (i.e., nature of the infectious disease(s) and/or anti-infective treatment studied), (2) data source(s), (3) year of publication, (4) types of information used in the main algorithm (i.e., algorithm targeting the condition of interest), (5) geographical scope, (6) years of study, (7) number of other health conditions targeted by an algorithm, (8) whether the article described the main algorithm in a reproducible way.

For validation studies, additional information collected were: (1) nature of reference standard, (2) recruitment criteria for the validation sample, (3) sample size, (4) study design, (5) performance parameters.

Based on the works by Benchimol et al. (23) and Widdifield et al. (24) we used a 35-items checklist to evaluate the quality of reported information for research identified as validation studies (23, 24). For each study, the expected number of items to be carried forward was calculated excluding uncertain and non-applicable items.

For studies using capture-recapture methods, we also collected: (1) complementary sources of cases used, (2) matching strategy between sources of information, (3) completeness estimator used, (4) whether the study has undertaken an assessment of the method's assumptions, (5) completeness estimate and its 95% confidence interval given in the manuscript or recalculated from the available data.

### 2.5. Performance indicators definitions

In the epidemiological area, sensitivity, specificity, positive predictive value, and negative predictive value are the four indicators commonly presented to describe the performance of a targeting algorithm.

Positive predictive value (PPV) informs us about the capacity of the algorithm to avoid the generation of falsely positive individuals among positive individuals and thus to discriminate only true cases. Negative predictive value (NPV), the counterpart of PPV, characterizes the capacity of the algorithm to avoid generating false negatives among negative subjects and therefore to discriminate individuals who are free of the disease. Sensitivity (Se) informs us about the ability of the algorithm to avoid the generation of false negatives among infected persons and therefore to identify all the cases. Specificity (Sp), counterpart of Se, characterizes the capacity of the algorithm to avoid generating false positive among non-infected subjects and thus informs us about the ability of the algorithm to identify all disease-free subjects.

Other performance indicators may be reported in epidemiological study such as likelihood ratio (25). Positive likelihood ratio is the ratio of the probability that the algorithm classifies a diseased person as positive (Se) to the probability that it classifies a disease-free person as positive (1-Sp). In contrast, the negative likelihood ratio is the ratio of the probability that the algorithm classifies a diseased person as negative (1-Se) to the probability that it classifies a disease-free person as negative (Sp).

## 3. Results

### 3.1. Studies selection

**Figure 1:**
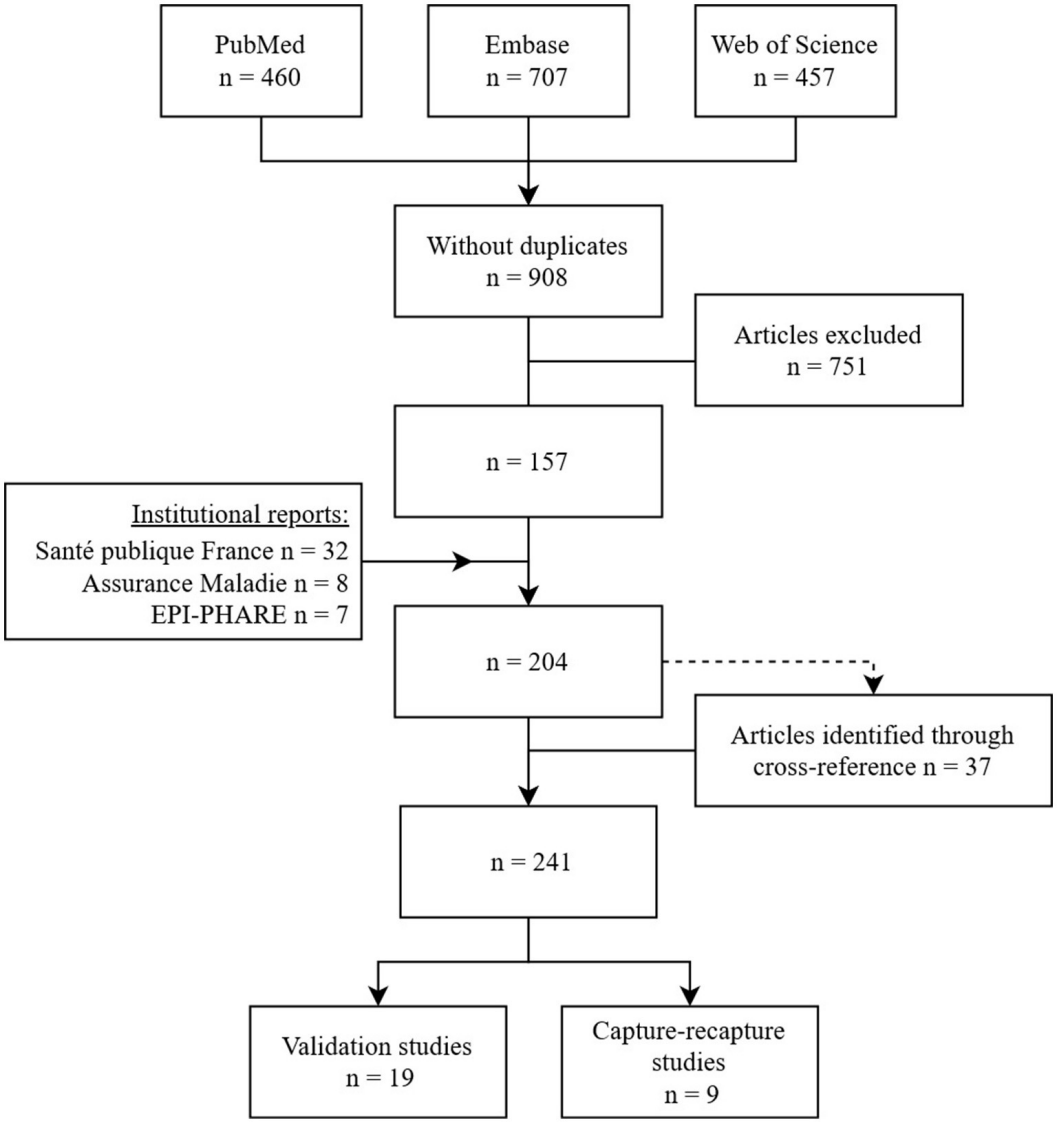
Flow diagram of articles selection studying epidemiology of infectious diseases using French medico-administrative databases published in English or French up to the end of 2021.

The methodical search resulted in the identification of 204 distinct studies. The analysis of their bibliographic references allowed the finding of 37 additional articles (Figure 1) among which five were not referenced by medical literature databases and four mentioned the infectious concept in their abstract only in very specific terms (abscess, dengue, malaria, and gastroenteritis). For the 28 other studies, the abstract made no mention to medico-administrative databases or referred to them with unusual terms.

From these 241 studies fulfilling the first three inclusion criteria (Supplementary 2), we finally identified 19 studies that evaluated the quality of definition algorithms and nine studies that estimated the completeness of French medico-administrative databases using a capture-recapture method. These studies looked at 16 different infectious diseases or infectious concepts including bone and joints infections, nosocomial infections, endocarditis, pneumonia, influenza and bronchiolitis, mucormycosis, herpetic and meningococcal invasive infections, gastro-enteritis and *Clostridium difficile*, urinary tract infections, hantavirus, malaria and dengue (Tables 1, **3**).

**Table 1:** Characteristics of validation studies.

**Study**	**Subject**	**Database**	**Algorithm evaluated**	**Reference standard**	**Source population criteria**	**Sample size**	**Approach^a^**	**Number of algorithms evaluated**	**Measures of accuracy**
Bitar et al. (37)	Mucormycosis	PMSI	ICD-10 codes	Medical expert review	Positive algorithm classification	179	3B	1	PPV = 0.36
Bounoure et al. (26)	mAGE	DCIR	Dispensed drugs + time lag between consultation and dispensation + patient's age	Patient's declaration	Targeted drug in the prescription	557	1	1	Se = 0.89 Sp = 0.89
Bounoure et al. (27)	mAGE	DCIR	Dispensed drugs + time lag between consultation and dispensation + patient's age	Patient's declaration	Targeted drug in the prescription	1 308	1	1	Se = 0.9 PPV = 0.82
de Lafforest et al. (59)	Urinary tract infections	PMSI	ICD-10 codes	Medical expert review	Algorithm classification, hospitalization unit	1 122	3A	1	Se = 0.95 Sp = 0.76 PPV = 0.70 NPV = 0.98
Dely et al. (25)	Preventable readmissions of CAP	PMSI	ICD-10 codes + time lag between hospitalisa tions + type of hospitalization admission	Medical expert review	Targeted ICD-10 code	415	2	5	Se = 0.31-0.5 Sp = 0.95-1 PPV = 0.36-0.66 PLR = 8.2-308
Gerbier et al. (60)	Nosocomial infections	PMSI	ICD-10 codes	Medical expert review	Surgical procedure	446	1 or 2	2	Se = 0.26-0.79 Sp = 0.66-1 PPV = 0.18-0.83 NPV = 0.94-0.97
				Reports to the center for the control of nosocomial infections	Stay in intensive care unit	1 499		4	Se = 0-0.59 Sp = 0.87 PPV = 0.09 NPV = 0.98
				Medical expert review and reports to the center for the control of nosocomial infections	Delivery in an obstetric unit	1 081		1	Se = 0.43 Sp = 0.786-1 PPV = 0-0.36 NPV = 0.88-0.98
Grammatico-Guillon et al. (61)	Vertebral osteomyelitis	PMSI	ICD-10 codes	Medical expert review	Positive algorithm classification	90	3B	1	PPV = 0.94
Grammatico-Guillon et al. (62)	Pneumococcal pneumonia	PMSI	ICD-10 codes	Medical expert review	Positive algorithm classification	45	3B	1	PPV = 0.82
				Laboratory results	positive pneumococcal sample	54	1 or 2		Se = 0.33 Sp = 1
Grammatico-Guillon et al. (63)	BJI	PMSI	ICD-10 codes + surgical procedure	Medical expert review	Positive algorithm classification	100	3B	1	PPV = 0.84
					Surgical procedure	205	1 or 2		Se = 0.95 Sp = 0.99 PPV = 0.98 NPV = 0.99
Grammatico-Guillon et al. (64)	Pediatric BJI	PMSI	ICD-10 codes + surgical procedure	Medical expert review	Algorithm classification, orthopedic fracture	398	3A	1	Se = 1 Sp = 0.8 PPV = 0.81 NPV = 1
Grammatico-Guillon et al. (65)	HKAI	PMSI	ICD-10 codes + surgical procedure	Medical expert review	Algorithm classification	1 010	3A	3	Se = 0.97-098 Sp = 0.71-0.95 PPV = 0.63-0.87 NPV = 0.98-0.99
Jones et al. (29)	Clostridium difficile infection	PMSI	ICD-10 codes	Laboratory results	Hospitalization	317 033	1 or 2	1	Se = 0.36 Sp = 1 PPV = 0.79 NPV = 1 κ= 0.49
Jouan et al. (66)	Herpes simplex encephalitis	PMSI	ICD-10 codes	Medical expert review	Algorithm classification, infection with neurological involvement	226	3A	1	PPV = 1 NPV = 1
Leclère et al. (28)	SSI	PMSI	ICD-10 codes + surgical procedure	Surveillance by the infection control team	Surgical procedure	4400	1 or 2	3	Se = 0.24-0.25 Sp = 0.98 PPV = 0.06-0.25 NPV = 0.98-1
Sahli et al. (67)	Various infections	PMSI	ICD-10 codes	Medical expert review	Positive algorithm classification	200	3B	2	PPV = 0.70-0.97
Soilly et al. (30)	RSV Bronchiolitis	PMSI	ICD-10 codes	Medical expert declaration	Hospitalization for bronchiolitis	302	1	1	Se = 0.55 Sp = 0.65 κ= 0.1
Sunder et al. (68)	Infective endocarditis	PMSI	ICD-10 codes	Medical expert review	Positive algorithm classification	198	3B	1	PPV = 0.87
					Surgical procedure	492	1 or 2		Se = 0.90 Sp = 1 PPV = 1 NPV = 0.99
Sunder et al. (69)	Infective endocarditis	PMSI	ICD-10 codes	Medical expert review	Positive algorithm classification	388	3B	1	PPV = 0.86
Tubiana et al. (70)	Oral streptococcal infective endocarditis	PMSI	ICD-10 codes	Medical expert review	Positive blood culture result for oral streptococci	130	1 or 2	1	Se = 0.54 PPV = 1

^a^see Figure 2 for a description of the different approaches. BJI, bone and joint infection; CAP, community-acquired pneumonia; DCIR, datamart de consummation inter-régime (national primary care consumption database); HKAI, hip or knee arthroplasty related infection; ICD-10, International Classification of Diseases 10th Revision; mAGE, medicalised acute gastroenteritis; NPV, negative predicted value; PLR, positive likelihood ratio; PMSI, programme de médicalisation des systems d'information (national hospital discharge summary database); PPV, positive predicted value; RSV, respiratory syncytial virus; Se, sensitivity; Sp, specificity; SSI, surgical site infection.

### 3.2. Validation studies

#### 3.2.1. Methodological framework

For 17 of the 19 studies identified, validation covered in-hospital events based on algorithms built from the hospital discharge database (PMSI) alone (Table 1). Only two studies used primary care reimbursement data (DCIR) to evaluate the performance of an algorithm identifying cases of medicalised acute gastroenteritis (26, 27).

Almost all the studies based on the PMSI (16 out of 17) used medical expert reviewing of patients' hospital records as gold standard. We also identified other methods used to constitute the gold standard, based on data from hospital microbiology laboratories or on data from a nosocomial infection control center. In the two studies that investigated primary care reimbursement data, the reference data was based on patients' self-report of their treatment indication (26, 27).

The inter-quantile range (IQR) of validation sample sizes varied from 193 to 1,028 individuals. One study was able to include 4,400 patients using data routinely collected by a hospital infection control team as the gold standard (28). One study used microbiology data as reference and was able to include up to 317,033 patients (29).

For each study, a median of three performance indicators were reported (IQR: 2–4). PPV was the most frequently reported indicator (17 out of 19 studies). NPV was reported in nine studies. Se of the algorithm was described in 14 studies and Sp was reported for 12 studies. Two other less common performance indicators were also identified. One study reported positive likelihood ratio, and two studies calculated the kappa coefficient, which is an indicator of the concordance between the classification made by the algorithm and the classification made by the gold standard (29, 30).

**Figure 2:**
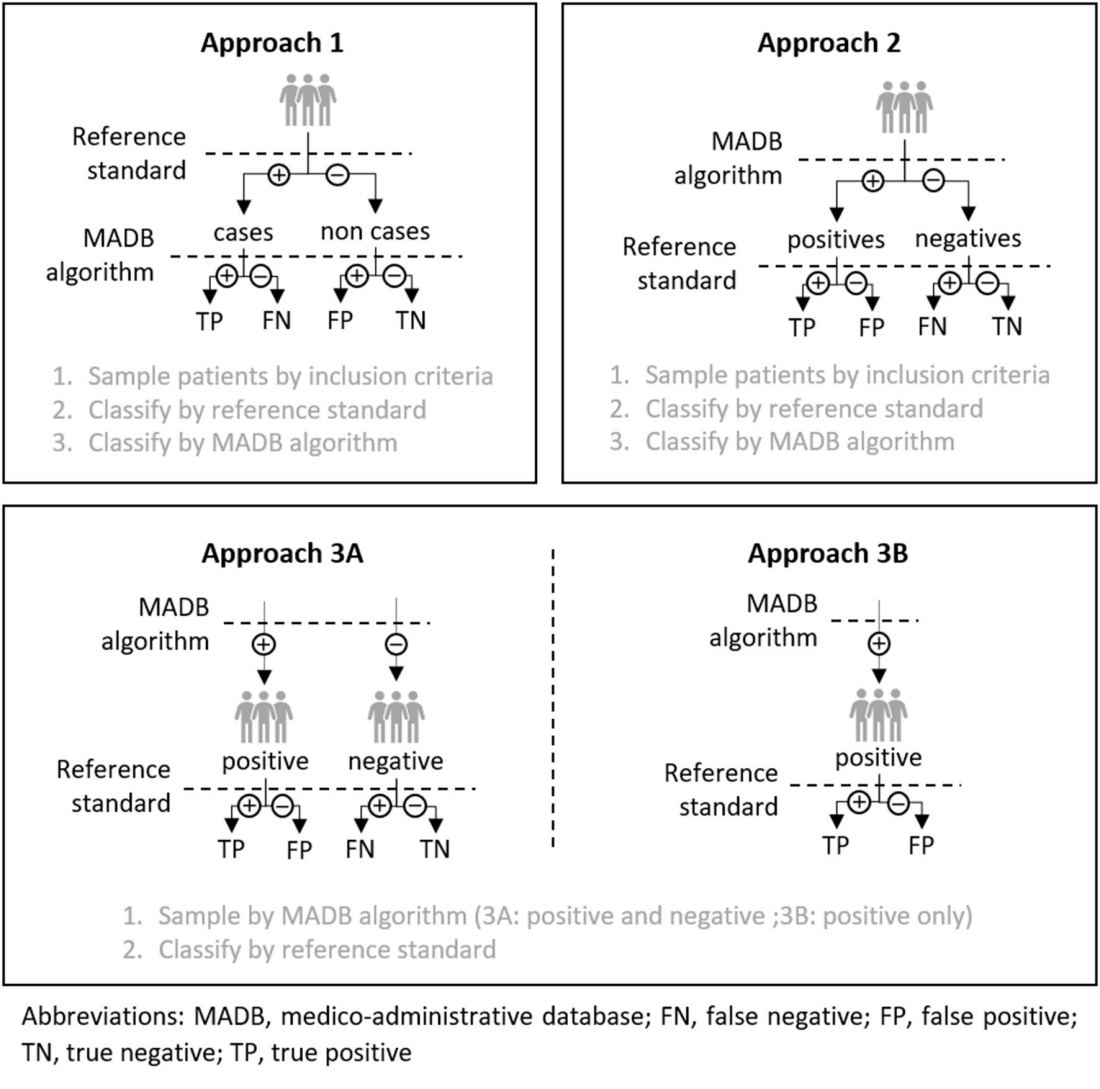
Approaches used to perform validation studies.

Based on the inclusion criteria of validation samples and according to the procedure steps of patients' classification into these samples, we determined four methodological approaches used to conduct the different studies (Figure 2).

Eleven studies used approaches 1 and 2 in which subjects were included and sampled independently of the classification results produced by the algorithm being evaluated. These first two approaches only differed by the order in which the algorithm and gold-standard classifications were applied. However, in seven of the 11 studies, the lack of clear indications about classification steps did not allow us to identify which of these two approaches has been applied. The calculation of all the principal performance parameters of the algorithm (Se, Sp, PPV, and NPV) is theoretically possible for both approaches assuming the sampling modes preserve the prevalence of the disease with respect to the inclusion criteria.

In approach 3A, used by four studies, sampling was carried out according to the result of the classification algorithm. This approach results in the constitution of two samples (patients classified as positive and patients classified as negative by the algorithm) whose sizes were chosen arbitrarily. This approach allows the calculation of positive and negative predictive values of the algorithm but should not be used to calculate Se and Sp (31) Indeed, the arbitrary choice of the number of positive and negative patients in the validation sample is likely to distort the “natural” ratio of these marginal distributions, leading to a bias in the estimation of Se and Sp parameters (see Supplementary 3). The approach 3B, used in seven studies, differs in that only a group of positive patients is selected. Thus, it only allows an evaluation of the PPV of the algorithm.

We did not identify any studies that constituted a validation sample by selecting an arbitrary number of cases and non-cases as defined by gold standard classification. This method leads to the same type of issue as methods 3A and 3B since it leads to a distortion of the natural ratio of the disease prevalence. Thus, this method would not allow the calculation of the predictive values of the algorithm but only of its Se and Sp.

#### 3.2.2. Quality of reported information

For all the studies (*n* = 19), out of the 35-items checklist a median of 56% of the expected items were reported (Q1-Q3, 40-65%) (Table 2). These statistics varied with the objectives of each study. For the 8 studies with algorithm validation as primary objective, a median of 66% of items were reported (Q1–Q3, 62–8%), compared with a median of 40% of items reported (Q1–Q3, 28–49%) for the studies where validation was a secondary objective.

**Table 2:** Evaluation of validation studies [35-items checklist based on the works by Benchimol et al. (23) and Widdifield et al. (24)].

**Section, criteria**	**Yes**	**No**	**Uncertain**	**NA**
**Title, Keywords, Abstract**				
1 - Identifies article as study of assessing diagnostic accuracy	13	6		
2 - Identifies article as study of administrative data	18	1		
**Introduction**				
3 - States disease identification and validation as one of the goals of study	11	8		
**Methods**				
4 - Describes data sources	13	6		
5 - Describes medico-administrative algorithm	19	0		
6 - Describes inclusion/exclusion criteria	19	0		
7 - Reports enrolment dates	14	5		
8 - Describes sampling method	11	8		
9 - Describe data collection for the reference standard	11	8		
10 - Use of a split sample for revalidation	0	19		
11 - Describes number, training or expertise of persons reading reference standard	10	9		
12 - Reports a measure of concordance if > 1 persons reading the reference standards	1	5		13
13 - Readers of the reference standard were blinded to the results of the classification by administrative data	1	6	12	
14 - Describes explicit methods for calculating or comparing measures of diagnostic accuracy and the statistical methods used to quantify uncertainty	4	15		
**Results**				
**Sample constitution**				
15 - Reports number of patient satisfying inclusion/exclusion criteria	19	0		
16 - Reports study flow diagram	3	16		
17 - If patients are sampled by reference standard, reports the number of records unable to link	1	0		18
18 - Reports number of missing/incomplete medical records and/or the number of patients unwilling to participate	6	13		
**Reports clinical and demographic characteristics of the validation sample**				
19 - Age	4	15		
20 - Sex	3	16		
21 - Comorbid conditions	2	17		
**Test results**				
22 - Presents cross tabulation of the results of the index tests by the results of the reference standard	7	12		
23 - Reports explicit pretest prevalence in the validation sample	6	9		4
24 - Describes the characteristics of misclassified patients (false-positive and/or false-negative)	6	13		
25 - Reports results for any subgroup (age, comorbidity, sex, location…)	6	13		
**Estimates**				
26 - Sensitivity	14	5		
27 - Specificity	12	7		
28 - Positive predictive value	17	2		
29 - Negative predictive value	9	10		
30 - Likelihood ratio	1	18		
31 - Kappa	2	17		
32 - Reports 95% confidence intervals	8	11		
33 - If PPV/NPV reported, number of case and control is not arbitrary defined?	16	0	1	2
34 – If Se/Sp reported, number of positive and negative is not arbitrary defined?	10	3	1	5
**Discussion**				
35 - Discusses the applicability of the study findings	18	1		

NA, not applicable; NPV, negative predicted value; PPV, positive predicted value; Se, sensitivity; Sp, specificity.

Almost two third of the studies were identified as validation studies of medico-administrative databases (12 out of 19). In all studies, the data sources referred to the medico-administrative data warehouse used, namely PMSI or DCIR. However, only 13 of the 19 studies identified and described the data sources used to constitute their gold standard.

All the studies reported a sufficiently detailed description of the algorithm evaluated and inclusion criteria for the validation sample to allow replication. In contrast, only half of the studies clearly stated the number and status of the persons who carried out the reference classification, and only one study explicitly stated that evaluators were blind to the results of the algorithm classification. Regarding the validation sample, only five studies reported at least one characteristic (age, sex, or comorbidity). Similarly, only six studies reported the specific characteristics of individuals misclassified by the algorithm. Eventually, only six studies reported having undertaken subgroup analyses to assess the influence of individual characteristics on the performance of the algorithm.

### 3.3. Capture-recapture studies

The nine studies that used a capture-recapture method were all based on PMSI data (Table 3). Except for one study using two complementary sources of information, all these studies matched PMSI data to a single other database.

**Table 3:** Characteristics of capture-recapture studies.

**Study**	**Subject**	**Database**	**Other sources**	**Matching strategy**	**Exhaustivity estimator**	**Database exhaustivity, 95%-CI**	**Assumption verifications**
Belchior et al. (36)	Hantavirus haemorrhagic fever with renal syndrome	PMSI	NRC for haemorrhagic fever	Age, sex, hospitalization year, place of residence	Chapman	0.37 (0.34-0.41)	NA
Bitar et al. (33)	Mucormycosis (deaths)	PMSI	Death certificates	NA	Sekar	0.43 (NA)	NA
Bitar et al. (37)	Mucormycosis	PMSI	NRC for invasive mycosis	Name, age, sex, hospitalization date	Chapman	0.52 (0.45-0.63)	Case definition
Dubos et al. (34)	Meningococcal invasive infection	PMSI	mandatory reporting database	Age, sex, place of residence, hospital identity, date of infection	Sekar or Chapman	0.73 (0.71-0.74)	Homogeneity
Kendjo et al. (32)	Malaria (deaths)	PMSI	Death certificates and NRC for malaria	Sex, date of death, place of death, age at death	Log-linear model	0.64 (0.60-0.67)	Case definition, Independence, Homogeneity
Loury et al. (38)	Severe influenza	PMSI	SpF surveillance system	Hospital identity, admission date (-1 day to + 7 days tolerance), sex, age (± 1 year tolerance)	Chapman	0.73 (0.72-0.74)	Homogeneity, Case definition (sensitivity analysis)
Molinié et al. (35)	Meningococcal invasive infection	PMSI	mandatory reporting database	Sex, age, place of residence, place of hospitalization, date of infection	Chapman	0.74 (0.69-0.79)	NA
Pivette et al. (39)	Severe influenza	PMSI	SpF surveillance system	Hospital identity, admission date (-1 day to + 7 days tolerance), sex, age (± 1 year tolerance)	Chapman	0.78 (0.77-0.79)	NA
Verrier et al. (40)	Dengue fever	PMSI	SpF surveillance system	Age (± 1 year tolerance), sex, place of residence, hospitalization place and date	Chapman	0.82 (0.78-0.86)	NA

PMSI, programme de médicalisation des systèmes d'information (national hospital discharge database); NRC, national reference center; SpF, Santé publique France (French public health institute); NA, not available.

These additional information sources can be categorized into four groups according to their origin and content. Data from the national register of death certificates (*CépiDC*) were used to estimate the number of deaths attributable to malaria and mucormycosis (32, 33). Two regional studies used mandatory declarations of meningococcal infection to adjust the disease incidence (34, 35). Three studies on mucormycosis, malaria, and haemorrhagic fevers with renal syndrome obtained clinical and biological data from the national reference centers (*Centers Nationaux de Réference*, CNR) related to these infections (32, 36, 37). Finally, two studies on severe influenza cases and one study on dengue fever benefited from the surveillance systems set up by the French public health agency (*Santé publique France*, SpF) (38–40).

To identify common patients between the PMSI and the complementary data sources, combinations of indirectly identifying data were used. Most frequently age, sex, and place of residence of the patients were used, along with location and dates of hospitalization. A single study included direct identifying data (surname and first name) (37).

The PMSI completeness was assessed by dividing the number of cases identified in this database by the total number of cases estimated using the capture-recapture method. This indicator varied according to the studied infection. For fever with renal syndrome and deaths related to mucormycosis the completeness was estimated at 37 and 43%, respectively (33, 36) In contrast, it was estimated that the PMSI recorded 82% of dengue cases on the Réunion island (40).

Of the nine studies reviewed, only four reported an assessment of assumptions involved in the capture-recapture method (validity of the case definition, completeness of case matching across sources, independence of sources and homogeneity of capture).

Validity of case definition was examined for mucormycosis by reviewing patient records and for malaria-related deaths by reviewing standardized hospital discharge summaries (32, 37). For severe influenza cases, sensitivity analysis with different VPP of the algorithm were carried out to investigate the impact of its validity on the estimation of the total number of cases (38).

The assumption of homogeneity of capture within each group implies that case identification within each source of information is equiprobable for all individuals regardless of their individual characteristics. To verify this hypothesis, one study on deaths associated with malaria and one study on severe influenza cases stratified their analysis according to three potential factors of heterogeneity: sex, age, and place of death for malaria and season, age, and place of residence for influenza (32, 38). Another study on invasive meningococcal infection also attempted to evaluate this hypothesis by comparing the distributions of cases between the two sources according to age and place of residence (34).

By linking three sources of information, the study on malaria-related deaths proposed to assess the dependency between these data sources by using log-linear models incorporating interaction terms between the different sources (32).

## 4. Discussion

This literature review identified epidemiological research on infectious diseases based on French medico-administrative databases, where validation efforts have been made, as well as the methods employed to that end. Showing strengths and limits of these approaches, this scoping review highlighted that both design of the validation study and characteristics of the validation sample are crucial to the quality of estimation of the algorithmic performance.

### 4.1. Validation studies

This review first highlights that to date, only a small fraction (8%) of the studies on infectious diseases based on medico-administrative data undertook to evaluate their infectious diseases definition algorithms. Hence, most infectious diseases commonly studied using medico-administrative data (e.g., influenza, COVID-19, meningitis, or HIV), do not yet have any algorithm assessed and validated.

The second finding of this review is that most studies focused exclusively on hospital discharge data (PMSI). Only two studies evaluating the performance of a gastroenteritis detection algorithm were based on primary care data (DCIR) (26, 27). Moreover, no validation study using both data sources jointly was identified. This observation could be explained by the relative simplicity of building a gold standard for evaluating an algorithm based on PMSI data alone. Indeed, as PMSI data are generated locally by each hospital, it is relatively straightforward for in-hospital experts to link patients' PMSI data with their clinical and biological data archived within the institution. However, validation of PMSI data alone is largely insufficient. By the end of 2021, research on infectious diseases based solely on PMSI represented only half of the studies published to date. This proportion is expected to decrease over time due to the expansion of access to SNDS beyond national health agencies to academic research.

The third lesson of this review is the importance of the validation study designs. Choosing a particular design is far from trivial since it determines the type of indicators that can be estimated and whether they can be generalized. Since post-test probabilities (PPV and NPV) are conditioned by the prevalence of the disease, they can only be estimated when the sampling of the validation panel is not based on the gold standard classification results. Indeed, if in the validation sample, the proportion of infected subjects (i.e., prevalence of infection) is higher than in the source population, PPV will be overestimated and NPV will be underestimated. For this reason, it is not possible to calculate these two parameters from a sample where the numbers of infected and non-infected patients are arbitrarily defined. Nevertheless, even when the study design allows for their calculation, these estimates should be interpreted with caution since they are conditioned by the prevalence of the infection in the source population. Thus, post-test probability estimators should always be considered conditional on the inclusion criteria chosen in the validation study. This dependence prohibits any generalization of these estimators. For instance, as the incidence of endocarditis in people with valve protheses is higher than in the general population, PPV and NPV of an algorithm targeting this infection estimated with a sample of people with valve protheses will not be generalisable to the general population.

While variability in post-test probabilities is usually well accepted, Se and Sp parameters are generally considered to be intrinsic properties of the test or algorithm being evaluated and therefore treated as constant values (41, 42). However, among the reviewed approaches of sample selection, some imply that the sampling of patients is done according to the classification results of the algorithm. By arbitrarily setting the ratio of the number of patients identified by the algorithm as infected and uninfected, a bias is introduced into the estimation of Se and Sp, whose magnitude depends on the real values of these parameters, the prevalence of the infection, and the chosen ratio of positive and negative patients (see Supplementary 3).

As with post-test probabilities, design of the validation study is not the only factor influencing Se and Sp. The concept of spectrum bias or spectrum effect is used to describe the variability in test performances depending on the characteristics of the validation samples (43). This effect, which is well characterized for biological tests of infectious diseases (44–46) should be particularly suspected when validating targeting algorithms in medico-administrative database. Unlike microbiological tests, which are based on biological markers, algorithms targeting infectious diseases in medico-administrative databases are based on medical parameters (e.g., diagnoses, anti-infectives prescriptions, or screening tests) which are the result of complex processes, interactions and decisions that are likely to vary broadly according to patient characteristics. For instance, a vulvar infection is more likely to be notified in the discharge summary of a pregnant or an immunocompromised woman than in the general population.

Thus, both design of the validation study and characteristics of the validation sample are critical for the quality of the estimates of algorithmic performance indicators. It therefore seems essential that authors of validation studies detail the design of their study and the estimation of valid performance indicators. Equally important, the precise reporting of the inclusion criteria and the description of the validation sample characteristics will allow to assess the scope of these performance indicators and consequently their transposability to future studies.

Eventually, it seems important to discuss the reuse of validated algorithm. In many cases, it appears that good performance parameters of an algorithm, in particular a high PPV, would alone justify its reuse and the robustness of the obtained results. However, even with satisfying performance, the few classification errors that an algorithm may generate are likely to induce significant bias in the results of a study. The magnitude and direction of this bias depend on the algorithm's performance, but also on its variability according to individual characteristics and the role of the algorithm in the study design (definition of an exposure/confounding, an outcome) (47). Thus, the reuse of algorithms for which performance indicators are available should always come along with a quantitative bias analysis to assess the impact of even minor misclassifications on the results of the research. Many quantitative bias analysis methods have been developed and implemented in most analysis software and are now extensively described (48).

### 4.2. Capture-recapture studies

Capture-recapture methods allow the simultaneous estimation of the infectious disease incidence and the completeness of data sources. However, these methods are limited by conditions of application which should be particularly questioned in the context of the use of medico-administrative database as information source (49).

The first criterion conditioning the validity of this method is that all the cases identified in the different sources are true cases. In the context of medico-administrative databases, this condition refers directly to the validity of the targeting algorithm and is therefore prone to be unsatisfied. Brenner demonstrated that in the case of a two-source model where only the medico-administrative database is affected by misclassification, and in absence of any correction for the status of misclassified patients, the total number of cases would always be overestimated by a factor equal to the inverse of the algorithm PPV (50). Two of the studies identified in this review took this issue into account through a systematic review of the medical records of all cases identified in the medico-administrative database to eliminate all false positive patients (32, 37). This strategy was feasible due to the scarcity inherent to the targeted events (mucormycosis and malaria-related deaths) resulting in a low volume of records to be reviewed (just over 200 in each of the two studies). Without necessarily proceeding to a systematic verification of cases identified, an evaluation of the PPV of the algorithm used in the medico-administrative database could allow correcting the overestimation induced thru misclassification by weighting the estimated total number of cases by the PPV value.

A second condition affecting the validity of the completeness estimators concerns the linkage of information sources. Indeed, capture-recapture implies that all cases common to the different sources are identified and that no case, in any source, is erroneously matched to a different case in another source. The relative impact of matching errors on the estimate of the total number of patients depends on the number of matching errors (erroneously matched cases and erroneously unmatched cases) and the actual number of cases shared by the information sources (see Supplementary 4) (51). Thus, if the overall matching error leads to an underestimation of the number of cases common to the databases, the estimate of the total number of patients will be overestimated and the completeness of the databases used will be underestimated. In this review, only one study was able to use a directly identifying variable (patient name) as a key to link the PMSI with the complementary data source (37). The other studies had to use a series of indirectly identifying variables to match patients. Three other studies also reported introducing some tolerance on the values of patients' age and dates of care to reduce the bias associated with data entry errors (38–40). This relaxation of the matching rules reduces the risk of missing a valid match but simultaneously increases the risk of false matches. This type of manipulation falls within the complex field of probabilistic matching methods that often prove to be indispensable when dealing with medico-administrative databases (52, 53). However, evaluating their results becomes particularly challenging in the context of approaches such as capture-recapture, since the number of cases to be matched is not known in advance. A sensitivity analysis should at least be carried out to assess the impact of linkage bias on the recapture estimators.

A third assumption underlying capture-recapture models is the independence of the sources of information involved. Dependence between two sources emerges when the presence of a case in one of them affects the probability of the case being present in the other source. A positive dependence results in an underestimation of the number of cases, while a negative dependence has the opposite effect (54). Capture-recapture studies included in this review all used the PMSI as source of information and linked it to additional sources coming from death certificates, national reference centers, mandatory reporting registers or surveillance networks. Given that the occurrence of an infectious disease case in the PMSI largely relies on diagnosis coded by hospital practitioners who are often responsible for notifying the other data sources, a positive dependency between the PMSI and the other data sources should be strongly suspected. The assessment of dependency between n sources always requires the involvement of at least an additional source (n+1). For this reason, it is strongly recommended to carry out capture-recapture studies based on at least 3 data sources (55). This literature review identified only one study that used three databases (32). This study on malaria mortality used two methods to assess and consider the dependence between sources in the estimation of the total number of deaths. The first method consists in making a contingency table of the presence of cases in the first two data sources using only the cases identified in the third source (55). Thus, this method provides the number of cases not recorded in the first two sources and enables evaluating their independence by a χ^2^ test or an odds ratio calculation. The second method consists to estimate the number of cases in the population using a log-linear model (49). Log-linear models have the advantage of allowing the integration of interaction terms to account for dependence between data sources. However, due to the impossibility to estimate the interaction of highest degree (i.e., the interaction involving all available sources simultaneously), log-linear methods cannot be used to evaluate dependency in a two sources capture-recapture study. Nevertheless, these methods can be used for sensitivity analysis in two sources capture-recapture study (56). Indeed, the integration of a parameter with a fixed value into the model allows to assess the impact of different degrees of dependency between sources on the estimated value of the total number of cases in the population.

A final constraint of capture-recapture methods concerns the homogeneity of captures within each source. In other words, the probability of inclusion within each data source must be identical for all cases, regardless of their individual characteristics (57). A first method to account for this potential bias is to stratify the recapture analysis on available variables. Two studies included in this review used this method as a sensitivity analysis by stratifying their analyses on patients' characteristics (32, 38). Another option is to consider covariates that are potentially sources of heterogeneity in the estimation of the total number of infectious disease cases in the population of interest by incorporating them into a log-linear model. It has been shown that this method also has the advantage of allowing the use of partially observed variables (i.e., variables not measured in all data sources) through the use of an expectation-maximization algorithm (58).

## 5. Conclusion

Despite many limitations, medico-administrative databases are sources of information that make possible the study of many health phenomena on considerable numbers of individuals. As their data supply is automated through health insurance systems, they constitute easily accessible and inexpensive sources of information that are highly complementary to more traditional sources of epidemiological data. For these reasons, their use for epidemiological purposes is expected to continue to grow. In the field of infectious diseases, they could therefore become a valuable resource for the monitoring of antimicrobial drug use, the surveillance of resistant or nosocomial infections, or the description of epidemic dynamics.

This literature review showed that despite its importance, quantitative evaluation of algorithms targeting infectious diseases in French medico-administrative databases is not yet a common practice for epidemiologists. This is undoubtedly linked to the fact that these evaluation studies, even though their seeming methodological simplicity, are in reality challenging to implement: constitution of a representative validation sample, definition and application of a reference classification, and above all regulatory and technical constraints linked to the matching of medico-administrative databases with other sources of information.

This literature review was also an opportunity to highlight the many risks of bias related to the underlying assumptions of these evaluation methods. These additional constraints should never be set aside by authors of evaluation studies but should be considered when planning their research work so that it can be compatible with analysis methods that allow the assessment of these assumptions or to take into account their violation. At the very least, these studies should include sensitivity analyses to assess the impact of breaching these assumptions on their estimates of performance parameters of the evaluated algorithms.

Despite all their limitations, medico-administrative databases are valuable sources of information for epidemiological research, especially if they are linked to other sources of information to enrich them with clinical and biological content. Linkages between medico-administrative databases and more conventional epidemiological databases should be encouraged and facilitated as they would allow both the implementation of more powerful observational studies and at the same time the evaluation and development of useful targeting algorithms when these medico-administrative databases are used alone.

## Author contributions

M-FT, KS, and LG-G contributed to the review conception and design. M-FT performed the literature search, study selection, data extraction, and wrote the first draft of the manuscript. All authors commented on previous versions of the manuscript, authors read, and approved the final manuscript.
