# Supplementary material for: Performance of French medico-administrative databases in epidemiology of infectious diseases: a scoping review
Source: Front Public Health. 2023 May 12;11:1161550. doi: 10.3389/fpubh.2023.1161550 (PMC10213695; doi:10.3389/fpubh.2023.1161550)
Supplement: Supplementary file 1 [file Data_Sheet_1.docx]

Supplementary Material

**Performance of French medico-administrative databases in epidemiology of infectious diseases: a scoping review**

Marc-Florent Tassi*, Nolwenn le Meur, Karl Stéfic, Leslie Grammatico-Guillon

*** Correspondence:** marc.tassi@etu.univ-tours.fr

# Supplementary 1: Search algorithm used in bibliographic biomedical databases

| **Concept** | | **Keyword** |
| --- | --- | --- |
| Infectious disease | | infect* OR virus OR viral* OR virol* OR bacter* OR sepsis* OR septic* OR vaccin* OR antibiot* OR antivir* OR fongiq* OR fungal* |
| **AND** | | |
| French medico-administrative databases | Name or acronyme | SNDS OR PMSI OR EGB OR SNIIRAM OR "SNIIR-AM" OR DCIR OR "système national des données de santé" OR "programme de médicalisation des systèmes" OR "échantillon généraliste de bénéficiaire*" OR "échantillon généraliste des bénéficiaires" OR "information inter-régime*" OR "information inter régime* "OR "consommation inter-régime*" OR "consommation inter régime* |
|  | **OR** | |
|  | **General reference** | **(France[Title/Abstract]) OR (French[Title/Abstract])** |
|  |  | **AND** |
|  |  | **"claim data*" OR "claims data*" OR "health care claim*" OR "health care data*" OR "health care system data*" OR "healthcare claim*" OR "healthcare data*" OR "healthcare system data*" OR "health data information system*" OR "health data system*" OR "health insurance information system*" OR "health record data*" OR "health system data*" OR "medical and administrative*" OR "medical-administrative*" OR "medico-administrative*" OR "discharge summary*" OR "discharge data*" OR "discharge system*" OR "hospital medical information*" OR "national hospital data*" OR "national hospitalization data*" OR "administrative data*" OR "insurance data*"** |
| **NOT** | | |
| Irrelevant terms | | **endoglucanase* OR "dendritic cell immunoreceptor*" OR "postmortem submersion interval*" OR "ginkgo biloba*" OR "strain EGB*"** |

# Supplementary 2: List of the 241 studies identified through the systematic literature search.

## Bibliography

Mageau A, Papo T, Ruckly S, Strukov A, van Gysel D, Sacre K, et al. Survival after COVID-19-associated organ failure among inpatients with systemic lupus erythematosus in France: a nationwide study. Ann Rheum Dis. 2022;81:569‑74.

2. Pol S, Fouad F, Lemaitre M, Rodriguez I, Lada O, Rabiega P, et al. Impact of extending direct antiviral agents (DAA) availability in France: an observational cohort study (2015-2019) of data from French administrative healthcare databases (SNDS). Lancet Reg Health Eur. 2022;13:100281.

3. Grave C, Gabet A, Empana JP, Puymirat E, Tuppin P, Danchin N, et al. Care management and 90-day post discharge mortality in patients hospitalized for myocardial infarction and COVID-19: A French nationwide observational study. Arch Cardiovasc Dis. 2021;115:37‑47.

4. Wyplosz B, Fernandes J, Goussiaume G, Moïsi J, Lortet-Tieulent J, Vainchtock A, et al. Adults at risk of pneumococcal disease in France. Infect Dis Now. 2021;51:661‑6.

5. Veziris N, Andréjak C, Bouée S, Emery C, Obradovic M, Chiron R. Non-tuberculous mycobacterial pulmonary diseases in France: an 8 years nationwide study. BMC Infectious Diseases. 2021;21:1165.

6. Tillard C, Chazard E, Faure K, Bartolo S, Martinot A, Dubos F. Burden of influenza disease in children under 2 years of age hospitalized between 2011 and 2020 in France. J Infect. 2021;84:145‑50.

7. Shen J, Bouée S, Aris E, Emery C, Beck EC. Long-Term Mortality and State Financial Support in Invasive Meningococcal Disease—Real-World Data Analysis Using the French National Claims Database (SNIIRAM). Infect Dis Ther. 2021;11:249‑62.

8. Prodel M, Finkielsztejn L, Roustand L, Nachbaur G, De Leotoing L, Genreau M, et al. Costs and mortality associated with HIV: a machine learning analysis of the French national health insurance database. J Public Health Res. 2021;11.

9. Le Vu S, Bertrand M, Jabagi J, Botton J, Drouin J, Baricault B, et al. Myocardite et péricardite après la vaccination Covid-19 [Internet]. EPI-PHARE; 2021 nov [cité 16 mars 2022]. Disponible sur: https://www.epi-phare.fr/rapports-detudes-et-publications/myocardite-pericardite-vaccination-covid19/

10. Epelboin S, Labrosse J, De Mouzon J, Fauque P, Gervoise-Boyer MJ, Levy R, et al. Obstetrical outcomes and maternal morbidities associated with COVID-19 in pregnant women in France: A national retrospective cohort study. PLoS Med. 2021;18:e1003857.

11. Cavalié P, Le Vu S, Maugat S, Berger-Carbonne A. Évolution de la consommation d’antibiotiques dans le secteur de ville en France 2010-2020. Quel est l’impact de la pandémie de Covid-19 ? Bulletin épidémiologique hebdomadaire. 2021;329‑35.

12. Covid-19 : efficacité vaccinale [Internet]. EPI-PHARE; 2021 oct [cité 16 mars 2022]. Disponible sur: https://www.epi-phare.fr/rapports-detudes-et-publications/impact-vaccination-covid-octobre-2021/

13. Tassi MF, Laurent E, Gras G, Lot F, Barin F, de Gage SB, et al. PrEP monitoring and HIV incidence after PrEP initiation in France: 2016-18 nationwide cohort study. J Antimicrob Chemother. 2021;76:3002‑8.

14. Ouattara E, Bruandet A, Borde A, Lenne X, Binder-Foucard F, Le-bourhis-zaimi M, et al. Risk factors of mortality among patients hospitalised with COVID-19 in a critical care or hospital care unit: analysis of the French national medicoadministrative database. BMJ Open Respiratory Research. 2021;8:e001002.

15. de Lafforest S, Magnier A, Vallée M, Bey E, Le Goux C, Saint F, et al. FUrTIHF: French urinary tract infections in healthcare facilities – five-year historic cohort (2014–2018). Journal of Hospital Infection. 2021;116:29‑36.

16. Conan Y, Laurent E, Belin Y, Lacasse M, Amelot A, Mulleman D, et al. Large increase of vertebral osteomyelitis in France: a 2010–2019 cross-sectional study. Epidemiology & Infection [Internet]. 2021 [cité 17 mars 2022];149. Disponible sur: https://www.cambridge.org/core/journals/epidemiology-and-infection/article/large-increase-of-vertebral-osteomyelitis-in-france-a-20102019-crosssectional-study/A0BB30322BEE38F0B54EF41A6AE83A60

17. Simon M, Thilly N, Pereira O, Pulcini C. Factors associated with the appropriateness of antibiotics prescribed in French general practice: a cross-sectional study using reimbursement databases. Clinical Microbiology and Infection [Internet]. 2021 [cité 21 mars 2022];0. Disponible sur: https://www.clinicalmicrobiologyandinfection.com/article/S1198-743X(21)00488-2/fulltext

18. Semenzato L, Botton J, Drouin J, Cuenot F, Dray-Spira R, Weill A, et al. Chronic diseases, health conditions and risk of COVID-19-related hospitalization and in-hospital mortality during the first wave of the epidemic in France: a cohort study of 66 million people. Lancet Reg Health Eur. 2021;8:100158.

19. Penso L, Dray-Spira R, Weill A, Pina Vegas L, Zureik M, Sbidian E. Association Between Biologics Use and Risk of Serious Infection in Patients With Psoriasis. JAMA Dermatology. 2021;157:1056‑65.

20. Grammatico-Guillon L, Miliani K, Banaei-Bouchareb L, Solomiac A, Sambour J, May-Michelangeli L, et al. A computerized indicator for surgical site infection (SSI) assessment after total hip or total knee replacement: The French ISO-ORTHO indicator. Infection Control & Hospital Epidemiology. 2021;1‑8.

21. Blanc E, Chaize G, Fievez S, Féger C, Herquelot E, Vainchtock A, et al. The impact of comorbidities and their stacking on short- and long-term prognosis of patients over 50 with community-acquired pneumonia. BMC Infectious Diseases. 2021;21:949.

22. Maitre T, Cottenet J, Godet C, Roussot A, Carime NA, Ok V, et al. Chronic pulmonary aspergillosis: prevalence, favouring pulmonary diseases and prognosis. European Respiratory Journal [Internet]. 2021 [cité 23 août 2021];58. Disponible sur: http://erj-ersjournals-com/content/58/2/2003345

23. Guillon A, Laurent E, Duclos A, Godillon L, Dequin PF, Agrinier N, et al. Case fatality inequalities of critically ill COVID-19 patients according to patient-, hospital- and region-related factors: a French nationwide study. ANNALS OF INTENSIVE CARE. 2021;11.

24. Demont C, Petrica N, Bardoulat I, Duret S, Watier L, Chosidow A, et al. Economic and disease burden of RSV-associated hospitalizations in young children in France, from 2010 through 2018. BMC Infectious Diseases. 2021;21:730.

25. Dalon F, Majed L, Belhassen M, Jacoud F, Bérard M, Lévy-Bachelot L, et al. Human papillomavirus (HPV) vaccine coverage rates (VCRs) in France: A French claims data study. Vaccine. 2021;39:5129‑37.

26. Semenzato L, Botton J, Drouin J, Cuenot F, Dray-Spira R, Weill A, et al. Covid-19 : facteurs de risques hospitalisation décès - 2ème vague [Internet]. EPI-PHARE; 2021 juill [cité 16 mars 2022]. Disponible sur: https://www.epi-phare.fr/rapports-detudes-et-publications/covid-19-facteurs-risques-2/

27. Penso L, Dray-Spira R, Weill A, Zureik M, Sbidian E. Psoriasis-related treatment exposure and hospitalization or in-hospital mortality due to COVID-19 during the first and second wave of the pandemic: cohort study of 1 326 312 patients in France*. British Journal of Dermatology. 2021;186:59‑68.

28. Jabagi J, Botton J, Bertrand M, Baricault B, Drouin J, Farrington P, et al. Vaccin Pfizer-BioNTech et événements cardiovasculaires graves [Internet]. EPI-PHARE; 2021 juill [cité 16 mars 2022]. Disponible sur: https://www.epi-phare.fr/rapports-detudes-et-publications/vaccin-pfizer-biontech-evenements-cardiovasculaires/

29. Gabet A, Grave C, Chatignoux E, Tuppin P, Béjot Y, Olié V. Characteristics, Management, and Case-Fatality of Patients Hospitalized for Stroke with a Diagnosis of COVID-19 in France. Neuroepidemiology. 2021;55:323‑30.

30. Weil-Olivier C, Taha MK, Emery C, Bouée S, Beck E, Aris E, et al. Healthcare Resource Consumption and Cost of Invasive Meningococcal Disease in France: A Study of the National Health Insurance Database. Infect Dis Ther. 2021;10:1607‑23.

31. Opatowski M, Brun-Buisson C, Touat M, Salomon J, Guillemot D, Tuppin P, et al. Antibiotic prescriptions and risk factors for antimicrobial resistance in patients hospitalized with urinary tract infection: a matched case-control study using the French health insurance database (SNDS). BMC Infectious Diseases. 2021;21:571.

32. Meyer A, Semenzato L, Zureik M, Weill A, Carbonnel F, Dray-Spira R. Risk of severe COVID-19 in patients treated with IBD medications: a French nationwide study. Alimentary Pharmacology & Therapeutics. 2021;54:160‑6.

33. Cheysson F, Brun-Buisson C, Opatowski L, Le Fouler L, Caserio-Schonemann C, Pontais I, et al. Outpatient antibiotic use attributable to viral acute lower respiratory tract infections during the cold season in France, 2010-2017. INTERNATIONAL JOURNAL OF ANTIMICROBIAL AGENTS. 2021;57.

34. Taine M, Offredo L, Drouin J, Toubiana J, Weill A, Zureik M, et al. Mandatory Infant Vaccinations in France During the COVID-19 Pandemic in 2020. Front Pediatr [Internet]. 2021 [cité 21 juill 2021];0. Disponible sur: https://www.frontiersin.org/articles/10.3389/fped.2021.666848/full

35. Iannelli A, Bouam S, Schneck AS, Frey S, Zarca K, Gugenheim J, et al. The Impact of Previous History of Bariatric Surgery on Outcome of COVID-19. A Nationwide Medico-Administrative French Study. Obes Surg. 2021;31:1455‑63.

36. Touat M, Brun-Buisson C, Opatowski M, Salomon J, Guillemot D, Tuppin P, et al. Costs and Outcomes of 1-year post-discharge care trajectories of patients admitted with infection due to antibiotic-resistant bacteria. J Infect. 2021;82:339‑45.

37. Simon M, Pereira O, Guillet-Thibault J, Hulscher MEJL, Pulcini C, Thilly N. Design of proxy indicators estimating the appropriateness of antibiotics prescribed by French dentists: a cross-sectional study based on reimbursement data. Antimicrob Agents Chemother. 2021;

38. Semenzato L, Botton J, Drouin J, Baricault B, Vabre C, Cuenot F, et al. Antihypertensive Drugs and COVID-19 Risk: A Cohort Study of 2 Million Hypertensive Patients. Hypertension. 2021;77:833‑42.

39. Piroth L, Cottenet J, Mariet AS, Bonniaud P, Blot M, Tubert-Bitter P, et al. Comparison of the characteristics, morbidity, and mortality of COVID-19 and seasonal influenza: a nationwide, population-based retrospective cohort study. Lancet Respir Med. 2021;9:251‑9.

40. Fosse-Edorh S, Mandereau-Bruno L. Hospitalisations pour Covid-19 au premier semestre 2020 chez les personnes traitées pharmacologiquement pour un diabète en France. Bulletin épidémiologique hebdomadaire. 2021;2‑8.

41. Bobbio A, Bouam S, Frenkiel J, Zarca K, Fournel L, Canny E, et al. Epidemiology and prognostic factors of pleural empyema. Thorax. 2021;76:1117‑23.

42. Bernard A, Cottenet J, Bonniaud P, Piroth L, Arveux P, Tubert-Bitter P, et al. Comparison of Cancer Patients to Non-Cancer Patients among COVID-19 Inpatients at a National Level. Cancers (Basel). 2021;13.

43. Taha MK, Weil-Olivier C, Bouée S, Emery C, Nachbaur G, Pribil C, et al. Risk factors for invasive meningococcal disease: a retrospective analysis of the French national public health insurance database. Hum Vaccin Immunother. 2021;1‑9.

44. Marchal C, Belhassen M, Guiso N, Jacoud F, Van Ganse E, Le Pannerer M, et al. Vaccination coverage rates for Diphtheria, Tetanus, Poliomyelitis and Pertussis booster vaccination in France between 2013 and 2017: Learnings from an analysis of National Health System Real-World Data. Vaccine. 2021;39:505‑11.

45. Dupuis C, Sabra A, Patrier J, Chaize G, Saighi A, Féger C, et al. Burden of pneumococcal pneumonia requiring ICU admission in France: 1-year prognosis, resources use, and costs. Crit Care. 2021;25:24.

46. Zeitoun JD, Duclos A, de Parades V, Lefèvre JH. Human papillomavirus vaccine uptake among men in France: a national time series analysis for 2015-2018. Hum Vaccin Immunother. 2020;16:3119‑22.

47. Tournaire G, Conte C, Perrot A, Lapeyre-Mestre M, Despas F. Vaccination during the First Diagnosis of Multiple Myeloma: A Cohort Study of the French National Health Insurance Database. Vaccines (Basel). 2020;8.

48. Pourcher V, Gourmelen J, Bureau I, Bouee S. Comorbidities in people living with HIV: An epidemiologic and economic analysis using a claims database in France. PLoS One. 2020;15:e0243529.

49. Kirchgesner J, Desai RJ, Beaugerie L, Schneeweiss S, Kim SC. Risk of Serious Infections With Vedolizumab Versus Tumor Necrosis Factor Antagonists in Patients With Inflammatory Bowel Disease. Clinical Gastroenterology and Hepatology [Internet]. 2020 [cité 29 juill 2021]; Disponible sur: https://www.sciencedirect.com/science/article/pii/S1542356520317262

50. Billioti de Gage S, Le Tri T, Dray-Spira R. Suivi de l’utilisation de la PrEP au VIH [Internet]. 2020 déc [cité 21 févr 2022]. Disponible sur: https://www.epi-phare.fr/rapports-detudes-et-publications/prep-vih-2020/

51. Robert J, Detournay B, Levant MC, Uhart M, Gourmelen J, Cohen JM. Flu vaccine coverage for recommended populations in France. Med Mal Infect. 2020;50:670‑5.

52. Cren PY, Bertrand N, Le Deley MC, Génin M, Mortier L, Odou P, et al. Is the survival of patients treated with ipilimumab affected by antibiotics? An analysis of 1585 patients from the French National hospital discharge summary database (PMSI). Oncoimmunology. 2020;9:1846914.

53. Thilly N, Pereira O, Schouten J, Hulscher MEJL, Pulcini C. Proxy indicators to estimate the appropriateness of medications prescribed by paediatricians in infectious diseases: a cross-sectional observational study based on reimbursement data. JAC-Antimicrobial Resistance [Internet]. 2020 [cité 25 nov 2021];2. Disponible sur: https://doi.org/10.1093/jacamr/dlaa086

54. Lamure S, Duléry R, Di Blasi R, Chauchet A, Laureana C, Deau-Fischer B, et al. Determinants of outcome in Covid-19 hospitalized patients with lymphoma: A retrospective multicentric cohort study. EClinicalMedicine. 2020;27:100549.

55. Fauchier L, Bisson A, Herbert J, Lacour T, Bourguignon T, Etienne CS, et al. Incidence and outcomes of infective endocarditis after transcatheter aortic valve implantation versus surgical aortic valve replacement. Clin Microbiol Infect. 2020;26:1368‑74.

56. Dupuis C, Bouadma L, Ruckly S, Perozziello A, Van-Gysel D, Mageau A, et al. Sepsis and septic shock in France: incidences, outcomes and costs of care. Ann Intensive Care. 2020;10:145.

57. Brouard C, Pillonel J, Boussac-Zarebska M, de Lédinghen V, Rachas A, Silvain C, et al. French hepatitis C care cascade: substantial impact of direct-acting antivirals, but the road to elimination is still long. BMC Infect Dis. 2020;20:759.

58. Kendjo E, Thellier M, Noël H, Jauréguiberry S, Septfons A, Mouri O, et al. Mortality from malaria in France, 2005 to 2014. Euro Surveill. 2020;25.

59. Blin P, Rouyer M, Guiard E, Zerbib F, Diquet B, Mégraud F, et al. Patterns of quadruple therapy use including bismuth for Helicobacter pylori eradication: A cohort study in the French national claims database. Therapie. 2020;

60. Escolano S, Mueller JE, Tubert-Bitter P. Accounting for indirect protection in the benefit-risk ratio estimation of rotavirus vaccination in children under the age of 5 years, France, 2018. Euro Surveill. 2020;25.

61. Thilly N, Pereira O, Schouten J, Hulscher ME, Pulcini C. Proxy indicators to estimate appropriateness of antibiotic prescriptions by general practitioners: a proof-of-concept cross-sectional study based on reimbursement data, north-eastern France 2017. Euro Surveill. 2020;25.

62. Sbidian E, Penso L, Herlemont P, Botton J, Baricault B, Semenzato L, et al. Covid-19 : antipaludéens et formes graves ou létales [Internet]. EPI-PHARE; 2020 juill [cité 4 mars 2021]. Disponible sur: https://www.epi-phare.fr/rapports-detudes-et-publications/covid-19-antipaludeens/

63. Pugnet G, Mounié M, Lapeyre-Mestre M, Moulis G, Sailler L. Couverture vaccinale anti grippale insuffisante dans l’artérite à cellules géantes. La Revue de Médecine Interne. 2020;41:446‑50.

64. Guillon A, Hermetet C, Barker KA, Jouan Y, Gaborit C, Ehrmann S, et al. Long-term survival of elderly patients after intensive care unit admission for acute respiratory infection: a population-based, propensity score-matched cohort study. Critical Care. 2020;24.

65. Bounoure F, Mouly D, Beaudeau P, Bentayeb M, Chesneau J, Jones G, et al. Syndromic Surveillance of Acute Gastroenteritis Using the French Health Insurance Database: Discriminatory Algorithm and Drug Prescription Practices Evaluations. Int J Environ Res Public Health. 2020;17.

66. Pivette M, Nicolay N, de Lauzun V, Hubert B. Characteristics of hospitalizations with an influenza diagnosis, France, 2012-2013 to 2016-2017 influenza seasons. Influenza Other Respir Viruses. 2020;14:340‑8.

67. Grave C, Boucheron P, Rudant J, Mikaeloff Y, Tubert-Bitter P, Escolano S, et al. Seasonal influenza vaccine and Guillain-Barré syndrome: A self-controlled case series study. Neurology. 2020;94:e2168‑79.

68. Ben Ghezala I, Mariet AS, Benzenine E, Bron AM, Baudin F, Daien V, et al. Incidence of acute postoperative endophthalmitis following macular surgery in France between 2006 and 2016. Acta Ophthalmol. 2020;98:e333‑8.

69. Riche VP, Schirr-Bonnans S, Cardaillac C, Le Thuaut A, Dert C, Mauduit N, et al. Hospital care pathway of women treated for Bartholin’s gland abscess and budget impact analysis of outpatient management: A national hospital database analysis. J Gynecol Obstet Hum Reprod. 2020;49:101689.

70. Maumus-Robert S, Debette S, Bérard X, Mansiaux Y, Tubert-Bitter P, Pariente A. Risk of Intracranial Aneurysm and Dissection and Fluoroquinolone Use: A Case-Time-Control Study. Stroke. 2020;51:994‑7.

71. Fauroux B, Hascoët JM, Jarreau PH, Magny JF, Rozé JC, Saliba E, et al. Risk factors for bronchiolitis hospitalization in infants: A French nationwide retrospective cohort study over four consecutive seasons (2009-2013). PLoS One. 2020;15:e0229766.

72. Costentin CE, Sogni P, Falissard B, Barbare JC, Bendersky N, Farges O, et al. Geographical Disparities of Outcomes of Hepatocellular Carcinoma in France: The Heavier Burden of Alcohol Compared to Hepatitis C. Digestive Diseases and Sciences. 2020;65:301‑11.

73. Luu M, Benzenine E, Barkun A, Doret M, Michiels C, Degand T, et al. Safety of first year vaccination in children born to mothers with inflammatory bowel disease and exposed in utero to anti-TNFα agents: a French nationwide population-based cohort. Aliment Pharmacol Ther. 2019;50:1181‑8.

74. Viriot D, Ndeikoundam Ngangro N, Lucas E, Dupin N, De Barbeyrac B, Bertolotti A, et al. Dépistage des IST bactériennes dans le secteur privé en France, 2006-2018. Bulletin épidémiologique hebdomadaire. 2019;634‑41.

75. Sunder S, Grammatico-Guillon L, Lemaignen A, Lacasse M, Gaborit C, Boutoille D, et al. Incidence, characteristics, and mortality of infective endocarditis in France in 2011. PLoS One. 2019;14:e0223857.

76. Pivette M, Loury P. Analyse de l’exhaustivité de la surveillance des grippes sévères en France métropolitaine, saison 2017-2018. Bulletin épidémiologique hebdomadaire. 2019;571‑2.

77. Gagneux-Brunon A, Pouvaret A, Maillard N, Berthelot P, Lutz MF, Cazorla C, et al. Acute kidney injury in infective endocarditis: A retrospective analysis. Med Mal Infect. 2019;49:527‑33.

78. de Lauzun V, Pivette M, Nicolay N, Hubert B. Caractéristiques des hospitalisations avec diagnostic de grippe, France métropolitaine, 2017-2018. Bulletin épidémiologique hebdomadaire. 2019;563‑70.

79. Barré T, Marcellin F, Di Beo V, Delorme J, Rojas Rojas T, Mathurin P, et al. Untreated alcohol use disorder in people who inject drugs (PWID) in France: a major barrier to HCV treatment uptake (the ANRS-FANTASIO study). Addiction. 2019;115:573‑82.

80. Fonteneau L, Barret AS, Levy-Bruhl D. Évolution de la couverture vaccinale du vaccin contre le papillomavirus en France - 2008-2018. Bulletin épidémiologique hebdomadaire. 2019;424‑30.

81. Dessauce C, Semenzato L, Rachas A, Barthélémy P, Lavin L, Comboroure JC, et al. Les antiviraux à action directe dans le traitement de l’hépatite C chronique : retour sur quatre ans de prise en charge par l’Assurance maladie (janvier 2014-décembre 2017). Bulletin épidémiologique hebdomadaire. 2019;502‑9.

82. Blondel C, Barret AS, Pelat C, Lucas E, Fonteneau L, Lévy-Bruhl D. Influence des facteurs socio-économiques sur la vaccination contre les infections à papillomavirus humain chez les adolescentes en France. Bulletin épidémiologique hebdomadaire. 2019;441‑50.

83. Colomb-Cotinat M, Assouvie L, Durand J, Daniau C, Leon L, Maugat S, et al. Epidemiology of Clostridioides difficile infections, France, 2010 to 2017. Euro Surveill. 2019;24.

84. Alari A, Cheysson F, Le Fouler L, Lanotte P, Varon E, Opatowski L, et al. Association of Pneumococcal Conjugate Vaccine Coverage With Pneumococcal Meningitis: An Analysis of French Administrative Areas, 2001-2016. Am J Epidemiol. 2019;188:1466‑74.

85. Verrier F, Etienne A, Vincent M, Vilain P, Lafont M, Mourembles G, et al. Sévérité de l’épidémie de dengue à La Réunion : données de surveillance des cas hospitalisés, avril 2017 à décembre 2018. Bulletin Epidémiologique Hebdomadaire. 2019;383‑9.

86. Dinh A, Le Monnier A, Emery C, Alami S, Torreton É, Duburcq A, et al. Predictors and burden of hospital readmission with recurrent Clostridioides difficile infection: a French nation-wide inception cohort study. Eur J Clin Microbiol Infect Dis. 2019;38:1297‑305.

87. Touat M, Opatowski M, Brun-Buisson C, Cosker K, Guillemot D, Salomon J, et al. A Payer Perspective of the Hospital Inpatient Additional Care Costs of Antimicrobial Resistance in France: A Matched Case-Control Study. Appl Health Econ Health Policy. 2019;17:381‑9.

88. Mageau A, Sacré K, Perozziello A, Ruckly S, Dupuis C, Bouadma L, et al. Septic shock among patients with systemic lupus erythematosus: Short and long-term outcome. Analysis of a French nationwide database. Journal of Infection. 2019;78:432‑8.

89. Lévy-Bruhl D, Fonteneau L, Vaux S, Barret AS, Antona D, Bonmarin I, et al. Assessment of the impact of the extension of vaccination mandates on vaccine coverage after 1 year, France, 2019. Eurosurveillance. 2019;24:1900301.

90. Mediamolle N, Mallet C, Aupiais C, Doit C, Ntika S, Vialle R, et al. Bone and joint infections in infants under three months of age. Acta Paediatr. 2019;108:933‑9.

91. Louis M, Cottenet J, Salmon-Rousseau A, Blot M, Bonnot PH, Rebibou JM, et al. Prevalence and incidence of kidney diseases leading to hospital admission in people living with HIV in France: an observational nationwide study. BMJ Open. 2019;9:e029211.

92. Creuzot-Garcher CP, Mariet AS, Benzenine E, Daien V, Korobelnik JF, Bron AM, et al. Is combined cataract surgery associated with acute postoperative endophthalmitis? A nationwide study from 2005 to 2014. Br J Ophthalmol. 2019;103:534‑8.

93. Septfons A, Goronflot T, Jaulhac B, Roussel V, De Martino S, Guerreiro S, et al. Epidemiology of Lyme borreliosis through two surveillance systems: the national Sentinelles GP network and the national hospital discharge database, France, 2005 to 2016. Euro Surveill. 2019;24.

94. Demessine L, Peyro-Saint-Paul L, Gardner EM, Ghosn J, Parienti JJ. Risk and Cost Associated With Drug-Drug Interactions Among Aging HIV Patients Receiving Combined Antiretroviral Therapy in France. Open Forum Infect Dis. 2019;6:ofz051.

95. Opatowski M, Tuppin P, Cosker K, Touat M, De Lagasnerie G, Guillemot D, et al. Hospitalisations with infections related to antimicrobial-resistant bacteria from the French nationwide hospital discharge database, 2016. Epidemiol Infect. 2019;147:e144.

96. Clémenty N, Carion PL, de Léotoing L, Lamarsalle L, Wilquin-Bequet F, Brown B, et al. Infections and associated costs following cardiovascular implantable electronic device implantations: a nationwide cohort study. Europace. 2018;20:1974‑80.

97. Baudin F, Benzenine E, Mariet AS, Bron AM, Daien V, Korobelnik JF, et al. Association of Acute Endophthalmitis With Intravitreal Injections of Corticosteroids or Anti-Vascular Growth Factor Agents in a Nationwide Study in France. JAMA Ophthalmol. 2018;136:1352‑8.

98. Hakimi Z, Ferchichi S, Aballea S, Odeyemi I, Toumi M, English M, et al. Burden of cytomegalovirus disease in allogeneic hematopoietic cell transplant recipients: a national, matched cohort study in an inpatient setting. Current Research in Translational Medicine. 2018;66:95‑101.

99. de Léotoing L, Barbier F, Dinh A, Breilh D, Chaize G, Vainchtock A, et al. French hospital discharge database (PMSI) and bacterial resistance: Is coding adapted to hospital epidemiology? Med Mal Infect. 2018;48:465‑73.

100. Maitre T, Cottenet J, Beltramo G, Georges M, Blot M, Piroth L, et al. Increasing burden of noninfectious lung disease in persons living with HIV: a 7-year study using the French nationwide hospital administrative database. Eur Respir J. 2018;52.

101. Galey C, Pouey J, Guillet A, Goria S, Mouly D. Détection d’épidémies de gastro-entérite aiguë médicalisée d’origine hydrique : Étude pilote concernant 7 départements de 7 régions françaises. Saint-Maurice: Santé publique France; 2018 sept p. 73 p.

102. de Léotoing L, Yazdanpanah Y, Finkielsztejn L, Chaize G, Vainchtock A, Nachbaur G, et al. Costs associated with hospitalization in HIV-positive patients in France. AIDS. 2018;32:2059‑66.

103. Couturier E, Abravanel F, Figoni J, Van Cauteren D, Septfons A, Lhomme S, et al. Surveillance de l’hépatite E en France, 2002-2016. Bulletin Epidémiologique Hebdomadaire. 2018;566‑74.

104. Laporte L, Hermetet C, Jouan Y, Gaborit C, Rouve E, Shea KM, et al. Ten-year trends in intensive care admissions for respiratory infections in the elderly. Ann Intensive Care. 2018;8:84.

105. Kirchgesner J, Lemaitre M, Carrat F, Zureik M, Carbonnel F, Dray-Spira R. Risk of Serious and Opportunistic Infections Associated With Treatment of Inflammatory Bowel Diseases. Gastroenterology. 2018;155:337-346.e10.

106. Couderc S, Lapeyre-Mestre M, Bourrel R, Paul C, Montastruc JL, Sommet A. Infectious risk of biological drugs vs. traditional systemic treatments in moderate-to-severe psoriasis: a cohort analysis in the French insurance database. Fundam Clin Pharmacol. 2018;32:436‑49.

107. Ndeikoundam Ngangro N, Viriot D, Lucas E, Boussac-Zarebska M, Lot F, Dupin N, et al. Relevance of healthcare reimbursement data to monitor syphilis epidemic: an alternative surveillance through the national health insurance database in France, 2011-2013. BMJ Open. 2018;8:e020336.

108. Mouly D, Goria S, Mounié M, Beaudeau P, Galey C, Gallay A, et al. Waterborne Disease Outbreak Detection: A Simulation-Based Study. Int J Environ Res Public Health. 2018;15.

109. Laurent E, Petit L, Maakaroun-Vermesse Z, Bernard L, Odent T, Grammatico-Guillon L. National epidemiological study reveals longer paediatric bone and joint infection stays for infants and in general hospitals. Acta Paediatr. 2018;107:1270‑5.

110. Laurent E, Gras G, Druon J, Rosset P, Baron S, Le-Louarn A, et al. Key features of bone and joint infections following the implementation of reference centers in France. Med Mal Infect. 2018;48:256‑62.

111. Rwagitinywa J, Lapeyre-Mestre M, Bourrel R, Sommet A. Generic antiretroviral drug use in HIV-infected patients: A cohort study from the French health insurance database. Therapie. 2018;73:257‑66.

112. van Cauteren D, Le Strat Y, Sommen C, Bruyand M, Tourdjman M, Jourdan Da Silva N, et al. Estimation de la morbidité et de la mortalité liées aux infections d’origine alimentaire en France métropolitaine, 2008-2013. Bulletin Epidémiologique Hebdomadaire. 2018;2‑10.

113. Devillers L, Sicsic J, Delbarre A, Le Bel J, Ferrat E, Saint Lary O. General Practitioner trainers prescribe fewer antibiotics in primary care: Evidence from France. PLoS One. 2018;13:e0190522.

114. Barbut F, Bouée S, Longepierre L, Goldberg M, Bensoussan C, Levy-Bachelot L. Excess mortality between 2007 and 2014 among patients with Clostridium difficile infection: a French health insurance database analysis. J Hosp Infect. 2018;98:21‑8.

115. Rivière M, Baroux N, Bousquet V, Ambert-Balay K, Beaudeau P, Jourdan Da Silva N, et al. Secular trends in incidence of acute gastroenteritis in general practice, France, 1991 to 2015. Eurosurveillance [Internet]. 2017 [cité 5 mars 2021];22. Disponible sur: https://www.eurosurveillance.org/content/10.2807/1560-7917.ES.2017.22.50.17-00121

116. Lamrani A, Tubert-Bitter P, Hill C, Escolano S. A benefit–risk analysis of rotavirus vaccination, France, 2015. Eurosurveillance. 2017;22:17.

117. Brouard C, Boussac-Zarebska M, Silvain C, Durand J, de Lédinghen V, Pillonel J, et al. Rapid and large-scale implementation of HCV treatment advances in France, 2007-2015. BMC Infect Dis. 2017;17:784.

118. Audureau E, Hua C, de Prost N, Hemery F, Decousser JW, Bosc R, et al. Mortality of necrotizing fasciitis: relative influence of individual and hospital-level factors, a nationwide multilevel study, France, 2007-12. Br J Dermatol. 2017;177:1575‑82.

119. Hakimi Z, Aballea S, Ferchichi S, Scharn M, Odeyemi IA, Toumi M, et al. Burden of cytomegalovirus disease in solid organ transplant recipients: a national matched cohort study in an inpatient setting. Transplant Infectious Disease. 2017;19:e12732.

120. Tubiana S, Blotière PO, Hoen B, Lesclous P, Millot S, Rudant J, et al. Dental procedures, antibiotic prophylaxis, and endocarditis among people with prosthetic heart valves: nationwide population based cohort and a case crossover study. BMJ. 2017;358:j3776.

121. Schwarzinger M, Baillot S, Yazdanpanah Y, Rehm J, Mallet V. Contribution of alcohol use disorders on the burden of chronic hepatitis C in France, 2008–2013: A nationwide retrospective cohort study. Journal of Hepatology. 2017;67:454‑61.

122. Miranda S, Chaignot C, Collin C, Dray-Spira R, Weill A, Zureik M. Human papillomavirus vaccination and risk of autoimmune diseases: A large cohort study of over 2million young girls in France. Vaccine. 2017;35:4761‑8.

123. Leblanc S, Blein C, Andremont A, Bandinelli PA, Galvain T. Burden of Clostridium difficile Infections in French Hospitals in 2014 From the National Health Insurance Perspective. Infect Control Hosp Epidemiol. 2017;38:906‑11.

124. Gallini A, Coley N, Andrieu S, Lapeyre-Mestre M, Gardette V. Effect of dementia on receipt of influenza vaccine: a cohort study in French older adults using administrative data: 2007-2012. Fundam Clin Pharmacol. 2017;31:471‑80.

125. Coly S, Vincent N, Vaissiere E, Charras-Garrido M, Gallay A, Ducrot C, et al. Waterborne disease outbreak detection: an integrated approach using health administrative databases. Journal of Water and Health. 2017;15:475‑89.

126. Loury P, Jones G, Chappert J, Pivette M, Hubert B. Analyse de l’exhaustivité et de la qualité de la surveillance des grippes sévères, 2009-2013. Saint-Maurice: Santé publique France; 2017 juill p. 59 p.

127. Hogan J, Pietrement C, Sellier-Leclerc AL, Louillet F, Salomon R, Macher MA, et al. Infection-related hospitalizations after kidney transplantation in children: incidence, risk factors, and cost. Pediatr Nephrol. 2017;32:2331‑41.

128. Rotily M, Abergel A, Branchoux S, Akremi R, de Léotoing L, Vainchtock A, et al. Disparités régionales des hospitalisations pour complication de l’hépatite chronique C en 2012. Sante Publique. 2017;Vol. 29:215‑27.

129. Delon F, Mayet A, Thellier M, Kendjo E, Michel R, Ollivier L, et al. Assessment of the French National Health Insurance Information System as a tool for epidemiological surveillance of malaria. J Am Med Inform Assoc. 2017;24:588‑95.

130. Pages F, Kurtkowiak B, Jaffar Bandjee M, Jaubert J, Domonte F, Traversier N, et al. Epidémiologie de la leptospirose à La Réunion, 2004-2015. Bulletin Epidémiologique Hebdomadaire. 2017;137‑46.

131. Pages F, Collet L, Henry S, Margueron T, Achirafi A, Bourhy P, et al. Leptospirose à Mayotte : apports de la surveillance épidémiologique, 2008-2015. Bulletin Epidémiologique Hebdomadaire. 2017;147‑56.

132. Moulis G, Lapeyre-Mestre M, Palmaro A, Sailler L. Infections in non-splenectomized persistent or chronic primary immune thrombocytopenia adults: risk factors and vaccination effect. J Thromb Haemost. 2017;15:785‑91.

133. Mallet V, Hamed K, Schwarzinger M. Prognosis of patients with chronic hepatitis B in France (2008–2013): A nationwide, observational and hospital-based study. Journal of Hepatology. 2017;66:514‑20.

134. Vainchtock A, Boudevin F, Chaize G, Jouaneton B, Durand-Zaleski I. Estimation de la fréquence et des coûts associés aux neutropénies fébrile en France : analyse de la base de données des hôpitaux (PMSI) 2010/2011. Journal de gestion et d’economie medicales. 2016;Vol. 34:17‑32.

135. Sahli L, Lapeyre-Mestre M, Derumeaux H, Moulis G. Positive predictive values of selected hospital discharge diagnoses to identify infections responsible for hospitalization in the French national hospital database. Pharmacoepidemiol Drug Saf. 2016;25:785‑9.

136. Daien V, Papinaud L, Gillies MC, Domerg C, Nagot N, Lacombe S, et al. Effectiveness and Safety of an Intracameral Injection of Cefuroxime for the Prevention of Endophthalmitis After Cataract Surgery With or Without Perioperative Capsular Rupture. JAMA Ophthalmol. 2016;134:810‑6.

137. Creuzot-Garcher C, Benzenine E, Mariet AS, de Lazzer A, Chiquet C, Bron AM, et al. Incidence of Acute Postoperative Endophthalmitis after Cataract Surgery: A Nationwide Study in France from 2005 to 2014. Ophthalmology. 2016;123:1414‑20.

138. Abergel A, Rotily M, Branchoux S, Akremi R, de Léotoing L, Vainchtock A, et al. Chronic hepatitis C: Burden of disease and cost associated with hospitalisations in France in 2012 (The HEPC-LONE study). Clin Res Hepatol Gastroenterol. 2016;40:340‑8.

139. Uhart M, Blein C, L’Azou M, Thomas L, Durand L. Costs of dengue in three French territories of the Americas: an analysis of the hospital medical information system (PMSI) database. Eur J Health Econ. 2016;17:497‑503.

140. Rambaud L, Galey C, Beaudeau P. Automated detection of case clusters of waterborne acute gastroenteritis from health insurance data - pilot study in three French districts. Journal of Water and Health. 2016;14:306‑16.

141. Raguideau F, Lemaitre M, Dray-Spira R, Zureik M. Association Between Oral Fluoroquinolone Use and Retinal Detachment. JAMA Ophthalmol. 2016;134:415‑21.

142. Fischer A, Castor C, Bonilla P, Noussitou M, Rolland P. Épidémie de gastro-entérites d’origine hydrique sur la commune de Laruns, Pyrénées-Atlantiques. Mars 2014. Saint-Maurice: Institut de veille sanitaire; 2016 mars p. 26 p.

143. Mouly D, Van Cauteren D, Vincent N, Vaissiere E, Beaudeau P, Ducrot C, et al. Description of two waterborne disease outbreaks in France: a comparative study with data from cohort studies and from health administrative databases. Epidemiol Infect. 2016;144:591‑601.

144. Le Meur N, Grammatico-Guillon L, Wang S, Astagneau P. Health insurance database for post-discharge surveillance of surgical site infection following arthroplasty. Journal of Hospital Infection. 2016;92:140‑6.

145. Dupouy Camet J, Touabet Azouzi N, Frealle E, van Cauteren D, Yera H, Moneret Vautrin A. Incidence de l’anisakidose en France. Enquête rétrospective 2010-2014. Bulletin Epidémiologique Hebdomadaire. 2016;64‑70.

146. Hua C, Sbidian E, Hemery F, Decousser JW, Bosc R, Amathieu R, et al. Prognostic factors in necrotizing soft-tissue infections (NSTI): A cohort study. Journal of the American Academy of Dermatology. 2015;73:1006-U184.

147. Fonteneau L, Ragot M, Parent du Châtelet I, Guthmann JP, Lévy-Bruhl D. The use of reimbursement data for timely monitoring of vaccination coverage: the example of human papillomavirus vaccine following public concerns about vaccine safety. BMC Public Health. 2015;15:1233.

148. Moulis G, Palmaro A, Sailler L, Lapeyre-Mestre M. Corticosteroid Risk Function of Severe Infection in Primary Immune Thrombocytopenia Adults. A Nationwide Nested Case-Control Study. PLoS One. 2015;10:e0142217.

149. Héquet D, Pouget N, Estevez JP, Robain M, Rouzier R. Age impact on human papillomavirus vaccination in France in 2014: A study from the National Health Insurance Database. Bull Cancer. 2015;102:892‑7.

150. Casez P, Fauconnier J, Jorgensen L, Gutterman EM, Gray S, Munson S, et al. Longitudinal DRG-based survey of all-cause and pneumococcal pneumonia and meningitis for inpatients in France (2005-2010). Med Mal Infect. 2015;45:446‑55.

151. Verger P, Cortaredona S, Pulcini C, Casanova L, Peretti-Watel P, Launay O. Characteristics of patients and physicians correlated with regular influenza vaccination in patients treated for type 2 diabetes: a follow-up study from 2008 to 2011 in southeastern France. Clinical Microbiology and Infection. 2015;21:930.e1-930.e9.

152. Le Monnier A, Duburcq A, Zahar JR, Corvec S, Guillard T, Cattoir V, et al. Hospital cost of Clostridium difficile infection including the contribution of recurrences in French acute-care hospitals. Journal of Hospital Infection. 2015;91:117‑22.

153. Grammatico-Guillon L, Baron S, Rosset P, Gaborit C, Bernard L, Rusch E, et al. Surgical Site Infection After Primary Hip and Knee Arthroplasty: A Cohort Study Using a Hospital Database. Infection Control & Hospital Epidemiology. 2015;36:1198‑207.

154. Fonteneau L, Ragot M, Guthmann JP, Lévy-Bruhl D. Use of health care reimbursement data to estimate vaccination coverage in France: Example of hepatitis B, meningitis C, and human papillomavirus vaccination. Rev Epidemiol Sante Publique. 2015;63:293‑8.

155. Van Cauteren D, Turbelin C, Fonteneau L, Hanslik T, De Valk H, Blanchon T. Physician practices in requesting stool samples for patients with acute gastroenteritis, France, August 2013-July 2014. Epidemiology and Infection. 2015;143:2532‑8.

156. Jouan Y, Grammatico-Guillon L, Espitalier F, Cazals X, François P, Guillon A. Long-term outcome of severe herpes simplex encephalitis: a population-based observational study. Crit Care. 2015;19:345.

157. ANSM, CNAM. Vaccins anti-HPV et risque de maladies auto-immunes [Internet]. ANSM; 2015 sept [cité 4 mars 2021]. Disponible sur: https://www.ameli.fr/fileadmin/user_upload/documents/rapport_final_ANSM_Cnamts_vaccins_anti_hpv_-_Septembre_2015.pdf

158. Van Cauteren D, De Valk H, Sommen C, King LA, Jourdan–Da Silva N, Weill FX, et al. Community Incidence of Campylobacteriosis and Nontyphoidal Salmonellosis, France, 2008–2013. Foodborne Pathogens and Disease. 2015;12:664‑9.

159. Schmidt A, Bénard S, Cyr S. Hospital Cost of Staphylococcal Infection after Cardiothoracic or Orthopedic Operations in France: A Retrospective Database Analysis. Surg Infect (Larchmt). 2015;16:428‑35.

160. Blein C, Gavazzi G, Paccalin M, Baptiste C, Berrut G, Vainchtock A. Burden of herpes zoster: the direct and comorbidity costs of herpes zoster events in hospitalized patients over 50 years in France. BMC Infect Dis. 2015;15:350.

161. Raffi F, Yazdanpanah Y, Fagnani F, Laurendeau C, Lafuma A, Gourmelen J. Persistence and adherence to single-tablet regimens in HIV treatment: a cohort study from the French National Healthcare Insurance Database. J Antimicrob Chemother. 2015;70:2121‑8.

162. Guthmann J, Fonteneau L, Collet M, Vilain A, Ragot M, Ben Boutieb M, et al. Couverture vaccinale hépatite B chez l’enfant en France en 2014 : progrès très importants chez le nourrisson, stagnation chez l’adolescent. Bulletin Epidémiologique Hebdomadaire. 2015;499‑504.

163. André V, Pot-Vaucel M, Cozic C, Visée E, Morrier M, Varin S, et al. Septic arthritis of the facet joint. Med Mal Infect. 2015;45:215‑21.

164. Botrel M, Fonteneau L, Boussac-Zarebska M, Parent du Chatelet I, Guthmann J, Levy-Bruhl D. Estimation des couvertures vaccinales à partir des données de l’Assurance maladie. Exemple de la vaccination contre le meningocoque C. Saint-Maurice: Institut de veille sanitaire; 2015 mai p. 4 p.

165. Moulis G, Lapeyre-Mestre M, Mahévas M, Montastruc JL, Sailler L. Need for an improved vaccination rate in primary immune thrombocytopenia patients exposed to rituximab or splenectomy. A nationwide population-based study in France. Am J Hematol. 2015;90:301‑5.

166. Sunder S, Grammatico-Guillon L, Baron S, Gaborit C, Bernard-Brunet A, Garot D, et al. Clinical and economic outcomes of infective endocarditis. Infect Dis (Lond). 2015;47:80‑7.

167. Pirard P, Goria S, Wakap SN, Galey C, Motreff Y, Guillet A, et al. No increase in drug dispensing for acute gastroenteritis after Storm Klaus, France 2009. Journal of Water and Health. 2015;13:737‑45.

168. Girard D, Antoine D, Che D. Epidemiology of pulmonary tuberculosis in France. Can the hospital discharge database be a reliable source of information? Med Mal Infect. 2014;44:509‑14.

169. Berrut G, Vainchtock A, Fernandez J, Bresse X, Baptiste C, Decker LD. Activité hospitalière et coûts liés à la prise en charge du zona en France : focus sur le zona ophtalmique. Gériatrie et Psychologie Neuropsychiatrie du Vieillissement. 2014;12:395‑401.

170. Bernier A, Delarocque-Astagneau E, Ligier C, Vibet MA, Guillemot D, Watier L. Outpatient antibiotic use in France between 2000 and 2010: after the nationwide campaign, it is time to focus on the elderly. Antimicrob Agents Chemother. 2014;58:71‑7.

171. Leclère B, Lasserre C, Bourigault C, Juvin ME, Chaillet MP, Mauduit N, et al. Matching bacteriological and medico-administrative databases is efficient for a computer-enhanced surveillance of surgical site infections: retrospective analysis of 4,400 surgical procedures in a French university hospital. Infect Control Hosp Epidemiol. 2014;35:1330‑5.

172. Hassen-Khodja C, Gras G, Grammatico-Guillon L, Dupuy C, Gomez JF, Freslon L, et al. Hospital and ambulatory management, and compliance to treatment in HIV infection: regional health insurance agency analysis. Med Mal Infect. 2014;44:423‑8.

173. Vandenesch A, Turbelin C, Couturier E, Arena C, Jaulhac B, Ferquel E, et al. Incidence and hospitalisation rates of Lyme borreliosis, France, 2004 to 2012. Eurosurveillance. 2014;19:20883.

174. Bitar D, Lortholary O, Le Strat Y, Nicolau J, Coignard B, Tattevin P, et al. Population-based analysis of invasive fungal infections, France, 2001-2010. Emerg Infect Dis. 2014;20:1149‑55.

175. Grammatico-Guillon L, Baron S, Gaborit C, Rusch E, Astagneau P. Quality assessment of hospital discharge database for routine surveillance of hip and knee arthroplasty-related infections. Infect Control Hosp Epidemiol. 2014;35:646‑51.

176. Birebent J, Dupouy J, Lempégnat J, Bourrel R, Bismuth M, Oustric S. Impact de la modification d’avis du Haut Conseil de la santé publique (HCSP) sur le taux de prescription des vaccins papillomavirus humains en région Midi-Pyrénées. Revue d’Épidémiologie et de Santé Publique. 2014;62:195‑9.

177. Septfons A, Gautier A, Brouard C, Bernillon P, Nicolau J, Larsen C. Prévalence, morbidité et mortalité associées aux hépatites B et C chroniques dans la population hospitalisée en France, 2004-2011. Bulletin Epidémiologique Hebdomadaire. 2014;202‑9.

178. Elkrief L, Chouinard P, Bendersky N, Hajage D, Larroque B, Babany G, et al. Diabetes mellitus is an independent prognostic factor for major liver-related outcomes in patients with cirrhosis and chronic hepatitis C. Hepatology. 2014;60:823‑31.

179. Beau AB, Hurault-Delarue C, Vidal S, Guitard C, Vayssière C, Petiot D, et al. Pandemic A/H1N1 influenza vaccination during pregnancy: a comparative study using the EFEMERIS database. Vaccine. 2014;32:1254‑8.

180. Beau AB, Hurault-Delarue C, Vial T, Montastruc JL, Damase-Michel C, Lacroix I. Safety of oseltamivir during pregnancy: a comparative study using the EFEMERIS database. BJOG: An International Journal of Obstetrics & Gynaecology. 2014;121:895‑900.

181. Fortin N, Gras Le Guen C, Picherot G, Guerin P, Moreau Klein A, Coste Burel M, et al. Caractéristiques des épidémies de bronchiolite dans l’agglomération nantaise, 2007-2012 : apport de différentes sources de données. Bulletin Epidémiologique Hebdomadaire. 2014;58‑64.

182. Lamarsalle L, Hunt B, Schauf M, Szwarcensztein K, Valentine WJ. Evaluating the clinical and economic burden of healthcare-associated infections during hospitalization for surgery in France. Epidemiol Infect. 2013;141:2473‑82.

183. Hanf M, Quantin C, Farrington P, Benzenine E, Hocine NM, Velten M, et al. Validation of the French national health insurance information system as a tool in vaccine safety assessment: application to febrile convulsions after pediatric measles/mumps/rubella immunization. Vaccine. 2013;31:5856‑62.

184. Bernard S, Mailles A, Stahl JP. Epidemiology of infectious encephalitis, differences between a prospective study and hospital discharge data. Epidemiol Infect. 2013;141:2256‑68.

185. Nuemi G, Astruc K, Aho S, Quantin C. [Comparing results of methicillin-resistant Staphylococcus aureus (MRSA) surveillance using the French DRG-based information system (PMSI)]. Rev Epidemiol Sante Publique. 2013;61:455‑61.

186. Ganry O, Bernin-Mereau AS, Gignon M, Merlin-Brochard J, Schmit JL. Human papillomavirus vaccines in Picardy, France: Coverage and correlation with socioeconomic factors. Revue d’Épidémiologie et de Santé Publique. 2013;61:447‑54.

187. Rotily M, Vainchtock A, Jouaneton B, Wartelle-Bladou C, Abergel A. How did chronic hepatitis C impact costs related to hospital health care in France in 2009? Clinics and Research in Hepatology and Gastroenterology. 2013;37:365‑72.

188. Lorgis L, Cottenet J, Molins G, Benzenine E, Zeller M, Aube H, et al. Outcomes after acute myocardial infarction in HIV-infected patients: analysis of data from a French nationwide hospital medical information database. Circulation. 2013;127:1767‑74.

189. Grammatico-Guillon L, Maakaroun-Vermesse Z, Baron S, Gettner S, Rusch E, Bernard L. Paediatric bone and joint infections are more common in boys and toddlers: a national epidemiology study. Acta Paediatr. 2013;102:e120-125.

190. Tuppin P, Choukroun S, Samson S, Weill A, Ricordeau P, Allemand H. Vaccination contre la grippe saisonnière en France en 2010 et 2011 : diminution des taux de couverture et facteurs associés. La Presse Médicale. 2012;41:e568‑76.

191. Coffinières E, Turbelin C, Riblier D, Aouba A, Levy-Bruhl D, Arena C, et al. Mumps: Burden of disease in France. Vaccine. 2012;30:7013‑8.

192. Nicolle E, Chinaud F, Pierre B, Escano G. Les prescriptions d’antibiotiques en ambulatoire en Alsace sur 22 mois entre 2008 et 2010. Pratiques et Organisation des Soins. 2012;43:81‑95.

193. Bitar D, Morizot G, Van Cauteren D, Dannaoui E, Lanternier F, Lortholary O, et al. Estimating the burden of mucormycosis infections in France (2005-2007) through a capture-recapture method on laboratory and administrative data. Rev Epidemiol Sante Publique. 2012;60:383‑7.

194. Grammatico-Guillon L, Baron S, Gettner S, Lecuyer AI, Gaborit C, Rosset P, et al. Bone and joint infections in hospitalized patients in France, 2008: clinical and economic outcomes. J Hosp Infect. 2012;82:40‑8.

195. Che D, Nicolau J, Bergounioux J, Perez T, Bitar D. Bronchiolite aiguë du nourrisson en France : bilan des cas hospitalisés en 2009 et facteurs de létalité. Archives de Pédiatrie. 2012;19:700‑6.

196. Beaudeau P, Rambaud L, Zeghnoun A, Corso M. Qualité de l’eau distribuée à Nantes et incidence des gastro-entérites aiguës. Saint-Maurice: Institut de veille sanitaire; 2012 juill p. 47 p.

197. Jones G, Taright N, Boelle PY, Marty J, Lalande V, Eckert C, et al. Accuracy of ICD-10 Codes for Surveillance of Clostridium difficile Infections, France - Volume 18, Number 6—June 2012 - Emerging Infectious Diseases journal - CDC. 2012 [cité 7 juill 2021]; Disponible sur: https://wwwnc.cdc.gov/eid/article/18/6/11-1188_article

198. Soilly AL, Ferdynus C, Desplanches O, Grimaldi M, Gouyon JB. Paediatric intensive care admissions for respiratory syncytial virus bronchiolitis in France: results of a retrospective survey and evaluation of the validity of a medical information system programme. Epidemiol Infect. 2012;140:608‑16.

199. Lanternier F, Dannaoui E, Morizot G, Elie C, Garcia-Hermoso D, Huerre M, et al. A Global Analysis of Mucormycosis in France: The RetroZygo Study (2005–2007). Clinical Infectious Diseases. 2012;54:S35‑43.

200. Grammatico-Guillon L, Thiercelin N, Mariani S, Lecuyer AI, Goudeau A, Bernard L, et al. [Study of hospitalizations for pneumococcal pneumoniae in Centre region, 2004-2008]. Rev Epidemiol Sante Publique. 2012;60:1‑8.

201. Dely C, Sellier P, Dozol A, Segouin C, Moret L, Lombrail P. [Preventable readmissions of « community-acquired pneumonia »: Usefulness and reliability of an indicator of the quality of care of patients’ care pathways]. Presse Med. 2012;41:e1-9.

202. Bonmarin I, Levy-Bruhl D. Analyse des données d’hospitalisation en France à partir du PMSI pendant la période pandémique 2009/2010. Saint-Maurice: Institut de veille sanitaire; 2012 janv p. 13 p.

203. Hubert B, Loury P, Ollivier R. Les hospitalisations pour grippe en service de réanimation dans la région des Pays-de-la-Loire (France), 2006-2011. Bulletin Epidémiologique Hebdomadaire. 2011;401‑4.

204. Rambaud L, Mouly D, Schmitt M, Kerrien F, Beaudeau P. Utilisation des données de l’Assurance maladie pour évaluer l’impact sanitaire d’une épidémie de gastro-entérites d’origine hydrique, Bourg-Saint-Maurice (Arc 1800), 2006. Bulletin Epidémiologique Hebdomadaire. 2011;339‑43.

205. Bouzbid S, Gicquel Q, Gerbier S, Chomarat M, Pradat E, Fabry J, et al. Automated detection of nosocomial infections: evaluation of different strategies in an intensive care unit 2000-2006. J Hosp Infect. 2011;79:38‑43.

206. Bounoure F, Beaudeau P, Mouly D, Skiba M, Lahiani-Skiba M. Syndromic surveillance of acute gastroenteritis based on drug consumption. Epidemiology & Infection. 2011;139:1388‑95.

207. Masson A, Ménetrey C, Garnier F, Bahans C, Fargeot A, Layadi M, et al. Incidence des pleuropneumopathies à pneumocoque en Limousin à l’ère de la vaccination. Archives de Pédiatrie. 2011;18:846‑9.

208. Perel C, Gallay A, Goulet V. La syphilis congénitale est-elle en recrudescence en France ? Enquête à partir du PMSI (2005-2007). Bulletin Epidémiologique Hebdomadaire. 2011;298‑300.

209. Tuppin P, Samson S, Weill A, Ricordeau P, Allemand H. Seasonal influenza vaccination coverage in France during two influenza seasons (2007 and 2008) and during a context of pandemic influenza A(H1N1) in 2009. Vaccine. 2011;29:4632‑7.

210. Tomas J, Lelièvre F, Bercelli P, Glanddier PY, Fanello S, Tuffreau F, et al. Hospital admissions related to influenza in France during the 2006/2007 epidemic. Revue d’Épidémiologie et de Santé Publique. 2011;59:159‑67.

211. Soumahoro MK, Boelle PY, Gauezere BA, Atsou K, Pelat C, Lambert B, et al. The Chikungunya Epidemic on La Reunion Island in 2005-2006: A Cost-of-Illness Study. Plos Neglected Tropical Diseases. 2011;5:e1197.

212. Fagot JP, Boutrelle A, Ricordeau P, Weill A, Allemand H. HPV vaccination in France: uptake, costs and issues for the National Health Insurance. Vaccine. 2011;29:3610‑6.

213. Gerbier S, Bouzbid S, Pradat E, Baulieux J, Lepape A, Berland M, et al. [Use of the French medico-administrative database (PMSI) to detect nosocomial infections in the University hospital of Lyon]. Rev Epidemiol Sante Publique. 2011;59:3‑14.

214. Gonzalez Chiappe S, Sarazin M, Turbelin C, Lasserre A, Pelat C, Bonmarin I, et al. Herpes zoster: Burden of disease in France. Vaccine. 2010;28:7933‑8.

215. Gueorguiev Penev D, Laurent E, Baron S, Diot E, Bastides F, de Gialluly C, et al. Borréliose de Lyme : recensement des cas adultes hospitalisés en Indre-et-Loire, à partir du PMSI (1999–2006). Revue d’Épidémiologie et de Santé Publique. 2010;58:339‑47.

216. Lerat A, Braillon A, Capron D, Tiberghien JP, Dubois G. [Prevention of hepatitis B transmission during the delivery in Picardy (2006)]. Presse Med. 2010;39:e182-187.

217. Vaux S, Brouard C, Fuhrman C, Turbelin C, Cohen J, Valette M, et al. Dynamique et impact de l’épidémie A(H1N1)2009 en France métropolitaine, 2009-2010. Bulletin Epidémiologique Hebdomadaire. 2010;259‑64.

218. Colin X, Berdeaux G, Lafuma A, Salvanet-Bouchara A, Kodjikian L. Inpatient costs of endophthalmitis evaluated for the whole of France. Appl Health Econ Health Policy. 2010;8:53‑60.

219. Braillon A, Nguyen-Khac E, Merlin J, Dubois G, Gondry J, Capron D. [HBsAg screening during pregnancy in the French province Picardy]. Gynecol Obstet Fertil. 2010;38:13‑7.

220. Tuppin P, Samson S, Weill A, Ricordeau P, Allemand H. Taux de couverture vaccinale contre la grippe en France en 2007–2008 : apport des données de remboursement du régime général. Médecine et Maladies Infectieuses. 2009;39:780‑8.

221. Bitar D, Van Cauteren D, Lanternier F, Dannaoui E, Che D, Dromer F, et al. Increasing Incidence of Zygomycosis (Mucormycosis), France, 1997-2006. Emerging Infectious Diseases. 2009;15:1395‑401.

222. Dubos F, Maréchal I, Tilmont B, Courouble C, Leclerc F, Martinot A. Incidence des infections invasives à méningocoque de l’enfant dans le Nord–Pas-de-Calais : intérêt et limites du programme de médicalisation des systèmes d’information (PMSI) pour la correction des données des déclarations obligatoires. Archives de Pédiatrie. 2009;16:984‑90.

223. Sabuncu E, David J, Bernède-Bauduin C, Pépin S, Leroy M, Boëlle PY, et al. Significant reduction of antibiotic use in the community after a nationwide campaign in France, 2002-2007. PLoS Med. 2009;6:e1000084.

224. Belchior E, Zeller H, Nicolau J, Vaillant V, Capek I. La fièvre hémorragique avec syndrome rénal en France métropolitaine de 2002 à 2007 : données du PMSI et du CNR. Bulletin Epidémiologique Hebdomadaire. 2009;233‑6.

225. Nicolay N, Gallay A, Michel A, Nicolau J, Desenclos JC, Semaille C. Reported cases of congenital syphilis in the French national hospital database. Euro Surveill. 2008;13.

226. Grammatico-Guillon L, Baron S, Rusch E, Lepage B, Surer N, Desenclos JC, et al. Epidemiology of vertebral osteomyelitis (VO) in France: analysis of hospital-discharge data 2002-2003. Epidemiol Infect. 2008;136:653‑60.

227. Mailles A, Vaillant V, Stahl JP. [Infectious encephalitis in France from 2000 to 2002: the hospital database is a valuable but limited source of information for epidemiological studies]. Med Mal Infect. 2007;37:95‑102.

228. Beaudeau P, Bounoure F. Évaluation épidémiologique d’indicateurs d’incidence des gastroentérites fondés sur les données de l’Assurance Maladie. Environnement, Risques & Santé. 2006;5:373‑82.

229. Baron S, Bonnemaison Gilbert E, Lanotte P, Despert F, Fourquet F, Goudeau A, et al. Bronchiolites, épidémiologie au Centre hospitalier régional universitaire de Tours, 1997-2005. Bulletin Epidémiologique Hebdomadaire. 2006;33‑4.

230. Bonmarin I, Ndiaye B, Seringe E, Levy-Bruhl D. Epidémiologie de la varicelle en France. Bulletin Epidémiologique Hebdomadaire. 2005;30‑1.

231. Suire-Saulnier A, Tabarly P. Évaluation de l’incidence de l’endocardite infectieuse en Limousin Poitou-Charentes et suivi bucco-dentaire de patients à risque. Revue Médicale de l’Assurance Maladie. 2004;35:243‑50.

232. Silvéra L, Flori Y, Slota L, Chinaud F, Weill A, Vallier N, et al. Coût et caractéristiques de la population traitée en ambulatoire par les antirétroviraux pour l’Assurance maladie en 2000 en France métropolitaine. Revue Médicale de l’Assurance Maladie. 2004;35:233‑42.

233. Allenbach D, Montagnier B, Souche A, Vallier N, Weill A, Chinaud F, et al. La population traitée par médicaments antituberculeux en 2003 : les données du régime général de l’Assurance maladie. Rev Med Ass Maladie. 2004;35:223‑32.

234. Lecadet J, Vialaret K, Vidal P, Baris B, Fender P. Mesure à l’échelle d’une région des effets d’un programme national d’information sur le bon usage des antibiotiques. Rev Med Ass Maladie. 2004;35:81‑9.

235. Fourquet F, Desenclos JC, Maurage C, Baron S. [Acute gastro-enteritis in children in France: estimates of disease burden through national hospital discharge data]. Arch Pediatr. 2003;10:861‑8.

236. Molinie F, Le Tourneau B, Ilef D. Estimation de l’exhaustivité du système de surveillance des infections à méningocoque dans le Nord-Pas-de-Calais, 1997-1998. Bulletin Epidémiologique Hebdomadaire. 2002;203‑5.

237. Haeghebaert S, Popoff M, Carlier J, Pavillon G, Delarocque-Astagneau E. Caractéristiques épidémiologiques du botulisme humain en France, 1991-2000. Bulletin Epidémiologique Hebdomadaire. 2002;57‑9.

238. Parjoie R, Cherrier C, Hérique A, Jeunehomme P, Weik J. Les antibiotiques. Analyse descriptive des prescriptions dans la région Nord-Est en 1998. Rev Med Ass Maladie. 2001;32:227‑34.

239. Lecadet J, Mora M, Baris B. Méningite à méningocoque : évaluer l’application de la chimioprophylaxie. Rev Med Ass Maladie. 2001;32:253‑6.

240. Chastagner M, Gault M, Coudene J, Desmerie C. Respect des contre-indications à la prescription de quinolones en médecine ambulatoire. Rev Med Ass Maladie. 2000;1:27‑32.

241. Desenclos JC, Rebière I, Letrillard L, Flahault A, Hubert B. Diarrhoea-related morbidity and rotavirus infection in France. Acta Paediatr Suppl. 1999;88:42‑7.

## Studies characteristics

|  | **Overall**  **N = 241** | **PMSI**  **N = 130** | | **DCIR**  **N = 41** | | **SNDS**  **N = 70** |
| --- | --- | --- | --- | --- | --- | --- |
| Scope of study, N (%) |  | |  | |  | |
| Pneumology | 46 (19%) | 34 (26%) | | 0 | | 12 (17%) |
| Gastro-enterology | 42 (17%) | 22 (17%) | | 13 (32%) | | 7 (10%) |
| Vaccines | 33 (14%) | 0 | | 13 (32%) | | 20 (29%) |
| Anti-infective drugs | 24 (10%) | 1 (0.8%) | | 13 (3%) | | 10 (14%) |
| STI | 14 (5.8%) | 6 (4.6%) | | 2 (5%) | | 6 (8.6%) |
| Invasive infection | 12 (5.0%) | 8 (6.2%) | | 0 | | 4 (5.7%) |
| HAI – microbial resistance | 11 (4.6%) | 10 (7.7%) | | 0 | | 1 (1.4%) |
| Neurology-dermatology | 11 (4.6%) | 11 (8.5%) | | 0 | | 0 |
| BJI | 11 (4.6%) | 11 (8.5%) | | 0 | | 0 |
| Vector-borne infection | 8 (3.3%) | 6 (4.6%) | | 0 | | 2 (2.9%) |
| Cardiology | 7 (2.9%) | 6 (4.6%) | | 0 | | 1 (1.4%) |
| Ophthalmology | 7 (2.9%) | 6 (4.6%) | | 0 | | 1 (1.4%) |
| Generalist | 7 (2.9%) | 2 (1.5%) | | 0 | | 5 (7.1%) |
| Other | 8 (3.3%) | 7 (5.4%) | | 0 | | 1 (1.4%) |
| Information used in the main algorithm, N (%) |  | |  | |  | |
| Hospital diagnosis | 142 (70%) | 130 (100%) | | 0 | | 32 (46%) |
| Drugs | 86 (36%) | 0 | | 41 (100%) | | 41 (59%) |
| Medical or chirurgical procedure | 18 (7.5%) | 16 (12%) | | 0 | | 2 (2.8%) |
| Laboratory analysis | 10 (4.1%) | 0 | | 1 (2.4%) | | 9 (13%) |
| Long term affection | 8 (3.3%) | 0 | | 1 (2.4%) | | 7 (10%) |
| Consultation | 5 (2.1%) | 0 | | 2 (4.9%) | | 3 (4.3%) |
| Number of information sources used in the main algorithm, N (%) |  | | | | | |
| 1 information source | 206 (85%) | 114 (88%) | | 37 (90%) | | 55 (79%) |
| 2 information sources | 27 (11%) | 16 (12%) | | 4 (10%) | | 7 (10%) |
| 3 information sources | 2 (0.8%) | 0 | | 0 | | 2 (2.9%) |
| 4 information sources | 5 (2.1%) | 0 | | 0 | | 5 (7.1%) |
| 5 information sources | 1 (0.4%) | 0 | | 0 | | 1 (1.4%) |
| Algorithm description (reproducibility), N (%) |  | |  | |  | |
| no description | 53 (22%) | 13 (10%) | | 19 (46%) | | 21 (30%) |
| partial description | 53 (22%) | 34 (26%) | | 1 (2.4%) | | 18 (26%) |
| complete description | 135 (56%) | 83 (64%) | | 21 (51%) | | 31 (44%) |
| Geographical scope, N (%) |  | |  | |  | |
| Pharmacy | 1 (0.4%) | 0 | | 1 (2.4%) | | 0 |
| Hospital(s) | 16 (6.6%) | 16 (12%) | | 0 | | 0 |
| Municipality(s) | 6 (2.5%) | 1 (0.8%) | | 5 (12%) | | 0 |
| Department(s) | 17 (7.1%) | 5 (3.8%) | | 10 (24%) | | 2 (2.8%) |
| Region(s) | 28 (23%) | 14 (11%) | | 7 (17%) | | 7 (10%) |
| France | 173 (72%) | 72 (70%) | | 18 (44%) | | 61 (87%) |
| Study duration, years (median, [Q1-Q3]) | 4 [2-6] | 4 [1 -7] | | 3 [2-6] | | 4.5 [2-6] |
| Time interval between last data and publication, years (median, [Q1-Q3]) | 3 [2-4] | 4 [2-5] | | 3 [2-4] | | 3 [1-4] |
| Other algorithms, N (%) | 118 (49%) | 63 (48%) | | 8 (20%) | | 47 (67%) |

Abbreviations: PMSI, Programme de médicalisation des systèmes d’information ; DCIR, Datamart de consommation inter-régime ; STI, Sexually transmitted infections ; HAI, Healthcare associated infections ; BJI, Bone and joint infections

## Trends in the use of French medico-administrative databases in the field of infectious diseases.


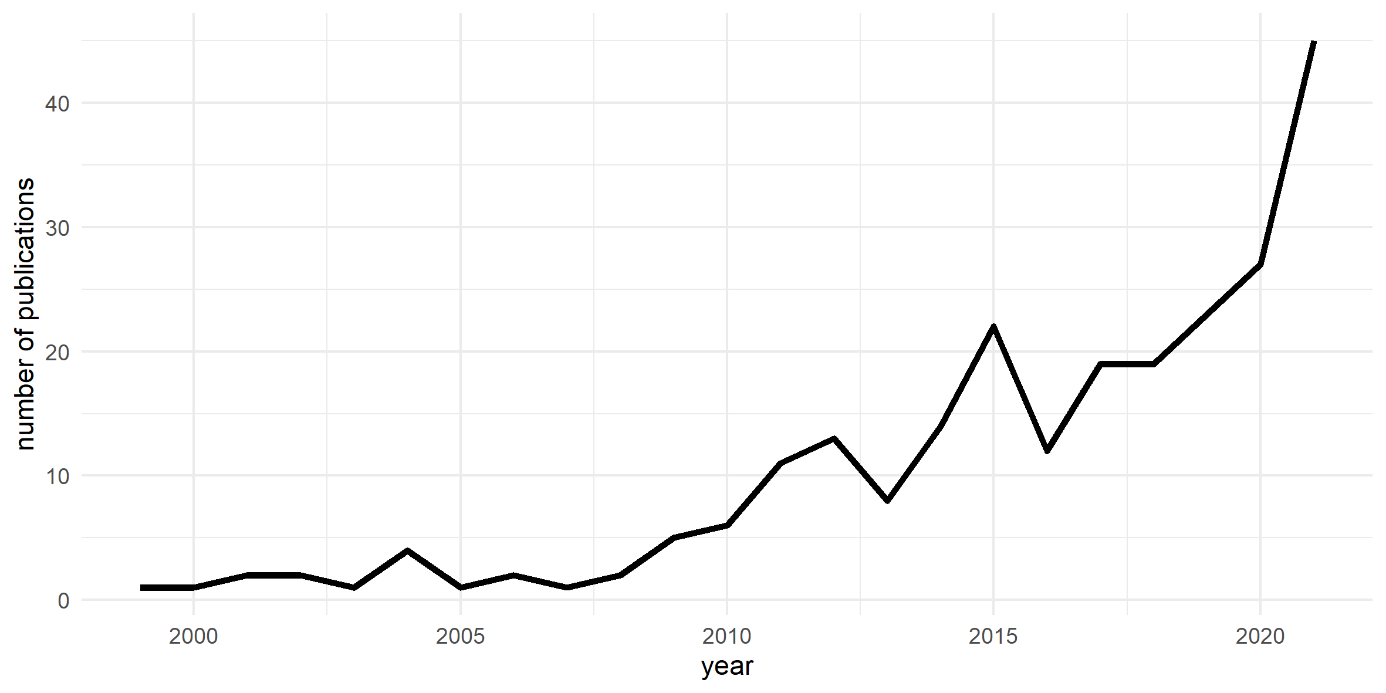


# Supplementary 3: link between the structure of the validation sample and magnitude of bias in the estimator of Se and Sp

The following graphs show the magnitude of the expected bias on the sensitivity and specificity values measured from the validation sample. The results are presented as a function of the parameters influencing this bias.

The true sensitivity (Se) and specificity (Sp) of the algorithm are presented on the x-axis and y-axis respectively.

Each row shows the results for a separate value of the natural ratio of positive patients over negative patients (Nr).

Each column presents the results for a value of the ratio (R) of the number of patients classified as positive to the number of patients classified as negative by the algorithm.

The bias (b) is calculated as the difference between the measured value of the parameter and its true value.

## Bias in measured Sensitivity


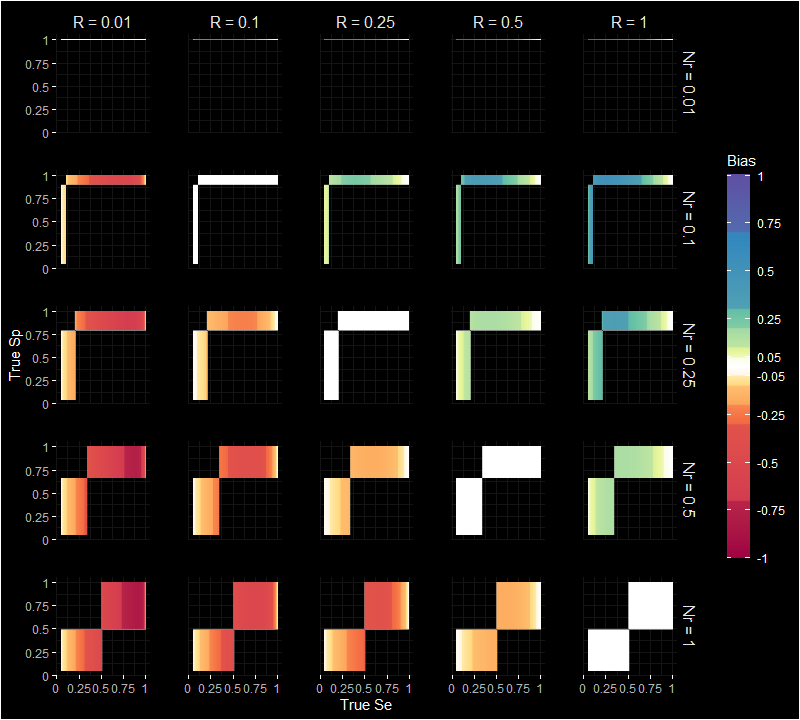


Bias, difference between measured sensitivity and true sensitivity; Nr, natural ratio of positive patients over negative patients; R, ratio of positive patients over negative patients as classified by the algorithm

## Bias in measured Specificity


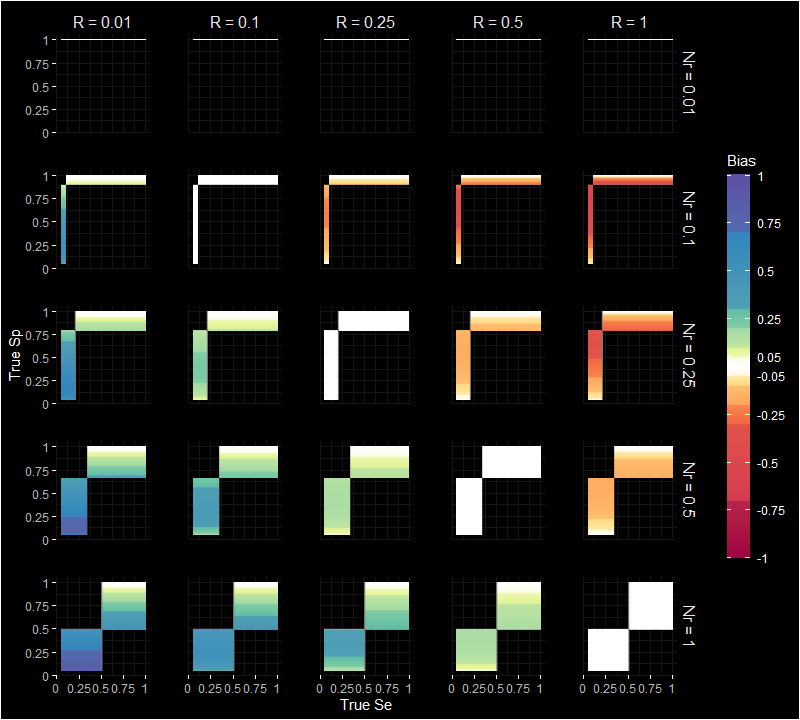


Bias, difference between measured specificity and true specificity; Nr, natural ratio of positive patients over negative patients; R, ratio of positive patients over negative patients as classified by the algorithm

# Supplementary 4: link between linkage error and bias magnitude in the Petersen estimator

We consider two information sources A and B whose respective sizes are $n_{A}$ et $n_{B}$. Let the presence of cases in source A be indexed by *i* (*i* = 0 for cases missing in source A, *i* = 1 for cases present in source A) and the presence of a case in source B be indexed by *j* (*j* = 0 for cases missing in source B, *j* = 1 for cases present in source B). The Petersen estimator of the population size can then be defined by the following equation:

| $\hat{N}= n_{10}+n_{01}+n_{11}+\frac{n_{10}n_{01}}{n_{11}}=\frac{n_{A}n_{B}}{n_{11}}$ | *1* |
| --- | --- |

The linkage bias *b* is then defined as the number of wrongly matched cases minus the number of wrongly unmatched cases. In the case of linkage error, the biased Petersen estimator is therefore equal to:

| $\hat{N}_{b}= {(n}_{10}-b)+{(n}_{01}-b)+{(n}_{11}+b)+\frac{{(n}_{10}-b){(n}_{01}-b)}{{(n}_{11}+b)}=\frac{n_{A}n_{B}}{{(n}_{11}+b)}$ | *2* |
| --- | --- |

The ratio between the biased Petersen estimator and the unbiased estimator is thus equal to:

| $\frac{\hat{N}_{b}}{\hat{N}}= \frac{n_{A}n_{B}}{{(n}_{11}+b)}\times\frac{n_{11}}{n_{A}n_{B}}= \frac{1}{1+\frac{b}{n_{11}}}$ | *3* |
| --- | --- |


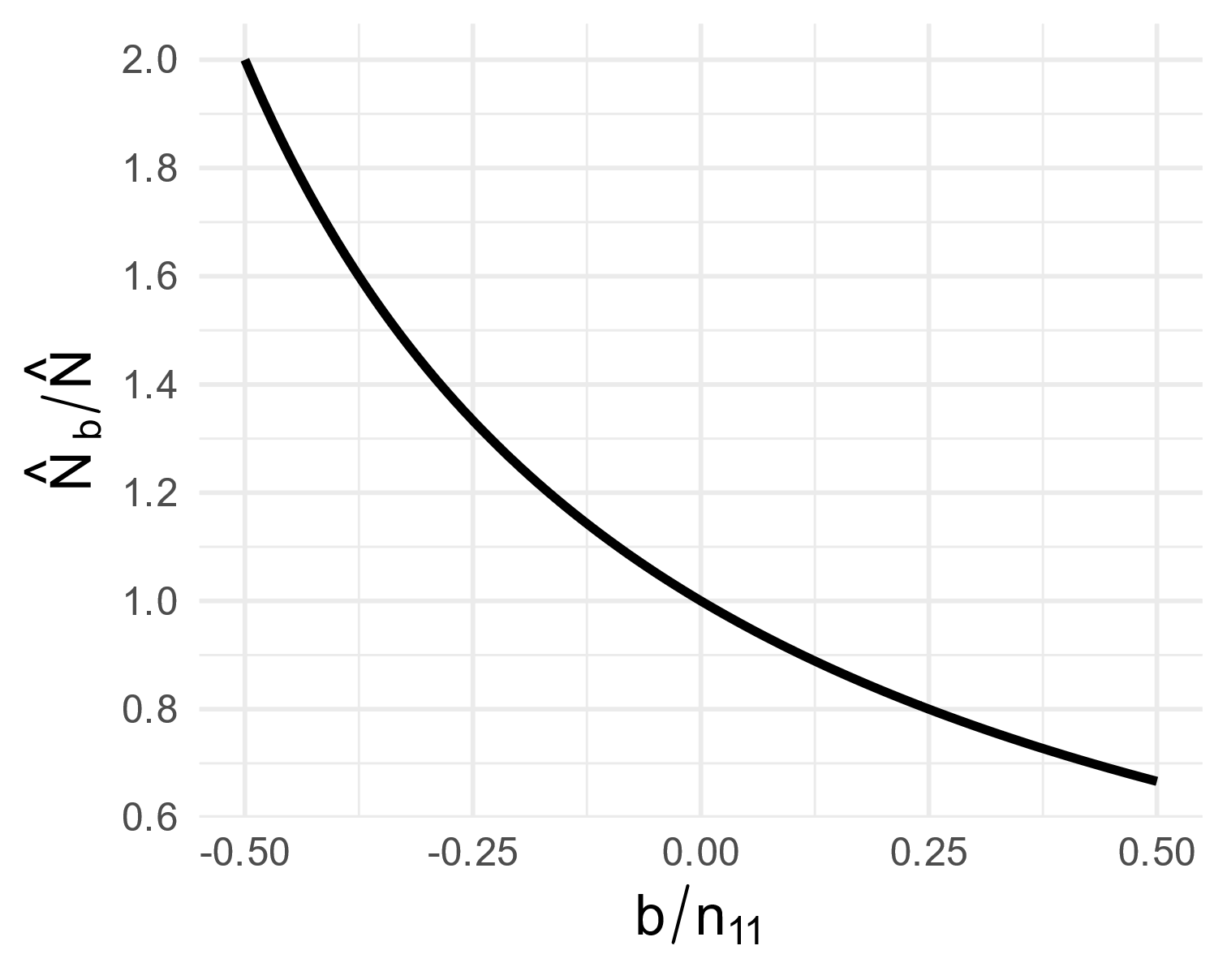


To illustrate this, we consider the results of the Pivette *et al* study on severe influenza as an example by applying different values of linkage bias to the data. In this study, 4 675 cases were identified in the PMSI database ($n_{A})$, 2 761 cases were identified in the surveillance programme database ($n_{B})$, and the linkage of these databases resulted in the identification of 2 157 common cases ($n_{11})$.

The table below presents values for the unbiased Petersen estimator $\hat{(N})$ and the estimation of PMSI exhaustivity under different values of linkage bias (*b)*

| *b* | $\hat{\boldsymbol{N}}$ | $\hat{\boldsymbol{N}}{\boldsymbol{-}\hat{\boldsymbol{N}}}_{\boldsymbol{b}}$ | PMSI exhaustivity |
| --- | --- | --- | --- |
| 500 | 7790 | 1806 | 0.60 |
| 300 | 6951 | 967 | 0.67 |
| 100 | 6275 | 291 | 0.75 |
| 50 | 6126 | 142 | 0.76 |
| 0 | 5984 | 0 | 0.78 |
| -50 | 5849 | -136 | 0.80 |
| -100 | 5719 | -265 | 0.82 |
| -300 | 5253 | -731 | 0.89 |
| -500 | 4858 | -1126 | 0.96 |
